# *DTYMK* is essential for genome integrity and neuronal survival

**DOI:** 10.1007/s00401-021-02394-0

**Published:** 2021-12-17

**Authors:** Jo M. Vanoevelen, Jörgen Bierau, Janine C. Grashorn, Ellen Lambrichs, Erik-Jan Kamsteeg, Levinus A. Bok, Ron A. Wevers, Marjo S. van der Knaap, Marianna Bugiani, Junmei Hu Frisk, Rita Colnaghi, Mark O’Driscoll, Debby M. E. I. Hellebrekers, Richard Rodenburg, Carlos R. Ferreira, Han G. Brunner, Arthur van den Wijngaard, Ghada M. H. Abdel-Salam, Liya Wang, Constance T. R. M. Stumpel

**Affiliations:** 1 Department of Clinical Genetics, Maastricht University Medical Centre+, 6229 ER Maastricht, The Netherlands; 2 Department of Human Genetics, Radboud UMC, 6525 GA Nijmegen, The Netherlands; 3 Department of Pediatrics, Màxima Medical Center, 5504 DB Veldhoven, The Netherlands; 4 Translational Metabolic Laboratory, Radboud UMC, 6525 GA Nijmegen, The Netherlands; 5 Department of Child Neurology, VUMC, 1105 AZ Amsterdam, The Netherlands; 6 Department of Neuropathology, VUMC, 1105 AZ Amsterdam, The Netherlands; 7 Department of Anatomy, Physiology and Biochemistry, Swedish University of Agricultural Sciences, 75007 Uppsala, Sweden; 8 Genome Damage and Stability Centre, University of Sussex, Brighton, BN1 9RH UK; 9 National Human Genome Research Institute, National Institutes of Health, Bethesda, MD 20892 USA; 10 Department of Clinical Genetics, Human Genetics and Genome Research Division, National Research Centre, Cairo, 12311 Egypt; 11 GROW-School for Oncology and Developmental Biology, 6229 ER Maastricht, The Netherlands; 12 MHENS School of Neuroscience, 6229 ER Maastricht, The Netherlands; 13 Donders Institute of Neuroscience, Radboud UMC, 6525 GA Nijmegen, The Netherlands

**Keywords:** DTYMK, dTMPK, Nucleotide metabolism, Zebrafish, Genome instability

## Abstract

Nucleotide metabolism is a complex pathway regulating crucial cellular processes such as nucleic acid synthesis, DNA repair and proliferation. This study shows that impairment of the biosynthesis of one of the building blocks of DNA, dTTP, causes a severe, early-onset neurodegenerative disease. Here, we describe two unrelated children with bi-allelic variants in *DTYMK,* encoding dTMPK, which catalyzes the penultimate step in dTTP biosynthesis. The affected children show severe microcephaly and growth retardation with minimal neurodevelopment. Brain imaging revealed severe cerebral atrophy and disappearance of the basal ganglia. In cells of affected individuals, dTMPK enzyme activity was minimal, along with impaired DNA replication. In addition, we generated *dtymk* mutant zebrafish that replicate this phenotype of microcephaly, neuronal cell death and early lethality. An increase of ribonucleotide incorporation in the genome as well as impaired responses to DNA damage were observed in *dtymk* mutant zebrafish, providing novel pathophysiological insights. It is highly remarkable that this deficiency is viable as an essential component for DNA cannot be generated, since the metabolic pathway for dTTP synthesis is completely blocked. In summary, by combining genetic and biochemical approaches in multiple models we identified loss-of-function of *DTYMK* as the cause of a severe postnatal neurodegenerative disease and highlight the essential nature of dTTP synthesis in the maintenance of genome stability and neuronal survival.

**Supplementary Information:**

The online version contains supplementary material available at 10.1007/s00401-021-02394-0.

## Introduction

**Fig. 1:**
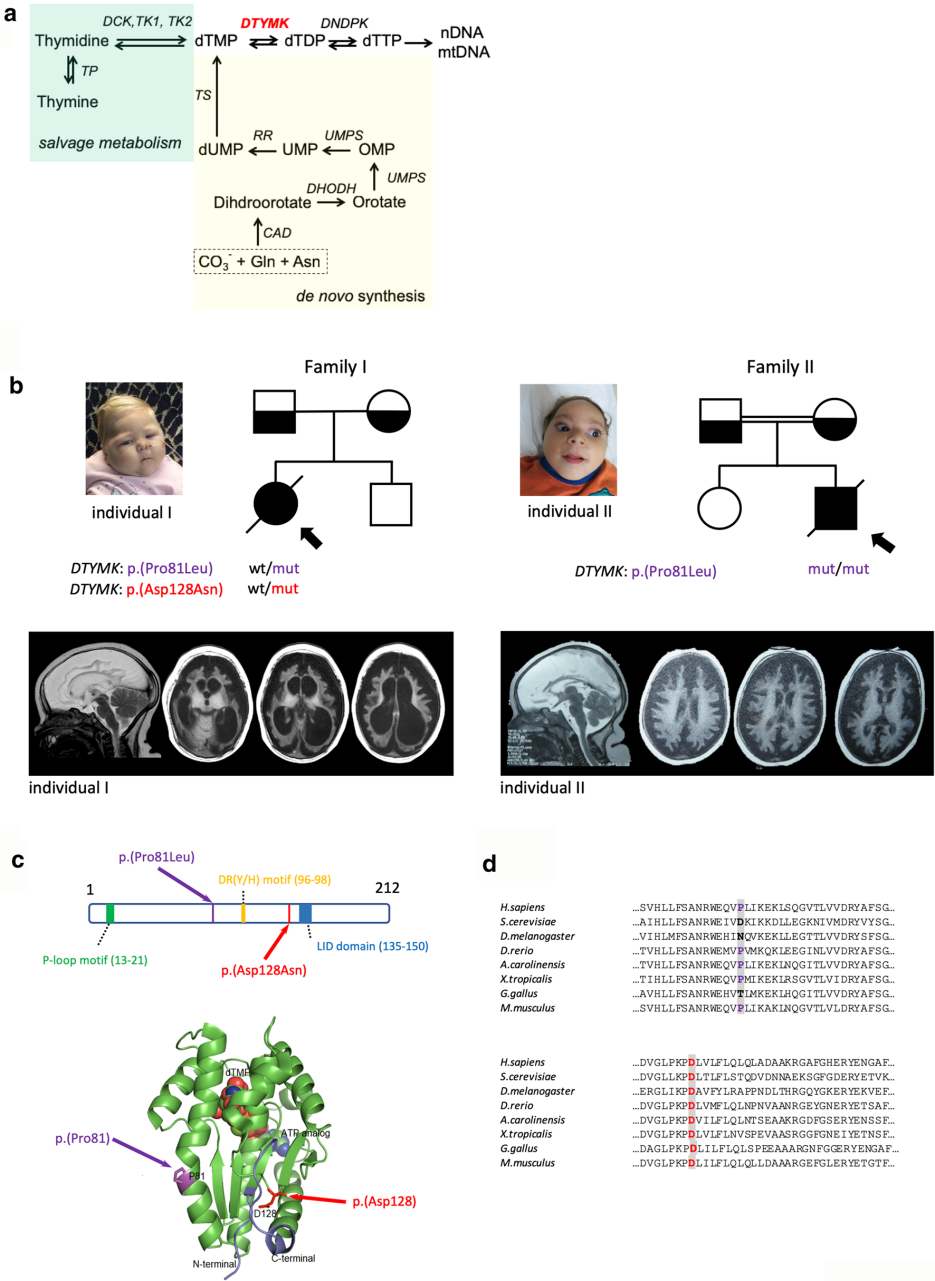
Description of variants and clinical phenotype. **a** Overview of the dTTP biosynthesis pathway. *DTYMK* (dTMPK) mediates the penultimate step of the generation of dTTP. Both pathways: salvage pathway and de novo pathway converge before the enzymatic defect in DTYMK. Abbreviations used: *DCK* deoxycytidine kinase; *TK1* thymidine kinase 1; *TK2* thymidine kinase 2 (mitochondrial); *DTYMK* deoxythymidine (monophosphate) kinase; *DNDPK* deoxynucleotide diphosphate kinase; *TS* thymidylate synthase; *RR* ribonucleotide reductase; *UMPS* uridine monophosphate synthetase; *CAD* carbamoyl-phosphate synthase 2, aspartate transcarbamylase and dihydroorotase; *TP* thymidine phosphorylase. **b** Description of two families carrying variants in *DTYMK.* Both families share the variant c.242C>T which is homozygous in individual II and indicated in purple. Family I is compound heterozygous and contains the additional variant c.382G>A, indicated in red. Brain MRI and pictures showing microcephaly in both affected individuals. The age of the individuals at which the pictures were taken are 9 months and 26 months for individual I and individual II, respectively. MRI images of individual I at age 6 months and of individual II at 12 months. Both subjects show severe atrophy of the cerebral hemispheres and basal ganglia, whereas the thalamus, brain stem and cerebellum appear normal. Individual I also shows pronounced dilation of the lateral ventricles. **c** Position of the observed variants in the primary protein sequence of dTMPK and relative position of known functional domains are shown in the upper panel. The variants are labelled in purple (p.(Pro81Leu)) and red (p.Asp128Asn). Known functional domains of DTYMK are: P-loop motif (indicated in green, residues 13–21), DR(Y/H)-motif (yellow, residues 96–98), LID domain (blue, residues 135–150) [26]. In the lower panel, the position of the variants in the 3D structure (PDB code 1e2f) are depicted in purple (p.Pro81Leu) and red (p.Asp128Asn). **d** Evolutionary conservation of regions in dTMPK containing the described variants. The protein sequences used in the alignment are: *H.sapiens*: ENSP00000304802; *S. cerevisiae*: YJR057W; *D. melanogaster*: FBpp0303030; *D. rerio*: ENSDARP00000068373; *A. carolinensis*: ENSACAP00000000660; *X. tropicalis*: ENSXETP00000044270; *G. gallus*: ENSGALP00000053279; *M. musculus*: ENSMUSP00000027503

Cellular processes such as proliferation and differentiation, DNA and RNA synthesis, repair and catabolism require a tightly regulated nucleotide metabolism. One of the central components here is dTTP (deoxythymidine triphosphate). Thymidine nucleotides are biosynthesized by reutilization of thymine and thymidine known as the salvage pathway (Fig. 1a). Alternatively, dTMP (deoxythymidine monophosphate) is formed by the de novo pathway, in which dUMP (deoxyuridine monophosphate) is converted to dTMP by thymidylate synthase (Fig. 1a). The de novo biosynthetic pathway is considered the predominant pathway in actively replicating cells, whereas non-cycling cells rely on the salvage metabolism [3].

In this study, we describe a dramatic neurological condition in two unrelated children (Table 1). Exome analysis identified variants in *DTYMK* (deoxythymidine kinase) that segregate with this condition. Functional studies in affected fibroblasts and in zebrafish substantiated the evidence of a causal relationship. *DTYMK* encodes dTMPK (deoxythymidine monophosphate kinase), a key enzyme in dTTP nucleotide metabolism (Fig. 1a). The thymidine de novo and salvage pathways converge at the point of the phosphorylation of dTMP to dTDP by dTMPK [13]. This makes dTMPK the bottleneck for dTTP biosynthesis (Fig. 1a).

**Table 1:** Overview of the most important clinical features of individual I and individual II

	Patient I	Patient II
Variant, genomic	Maternal: Chr2(GRCh37):g.242618013C>T	Maternal: Chr2(GRCh37):g.242619732G>A
	Paternal: Chr2(GRCh37):g.242619732G>A	Paternal: Chr2(GRCh37):g.242619732G>A
Variant, cDNA	Maternal: NM_012145.3:c.382G>A	NM_012145.3:c.242C>T
	Paternal: NM_012145.3:c.242C>T	
Variant, protein	Maternal: p.Asp128Asn; Paternal: p.Pro81Leu	p.Pro81Leu
Origin	The Netherlands	Egypt
Prenatal findings	Polyhydramnios, OFC at 36 weeks 10th centile	Slightly smaller head
Microcephaly	Yes	Yes
Development	No developmental progress	No developmental progress
Hearing	Normal	Normal
Vision	No tracking of objects, only light/dark	No tracking of objects
Sex	Female	Male
Birth weight, grams / SD	2645 / − 2.3 SD	2250 / − 1.8 SD
Seizures	Recurrent febrile seizures	myoclonic jerks
Weight, kg / age, months / SD	8,0 / 17 / − 2.6 SD	7.2 / 30 / − 4.0 SD
Height, cm / age, months / SD	58 / 16 / − 6.5 SD	68 / 26 / − 5.4 SD
Head circumference, cm / age, months, / SD	34 / 9 / − 7.6 SD	38 / 26 / − 7.4 SD
MRI brain	Severe atrophy of cerebral hemispheres and basal ganglia	Profound generalized volume loss cerebral cortex
	Brain stem and cerebellum appeared normal	Cerebellum unaffected
Other findings	Puffy body	puffy hands and feet, full cheeks
	Died at age 18 months	Died at age 32 months
		Microcytic hypochromic anemia and elevated liver enzymes
		Micropenis and undescended testis

This report is the second study showing an association between *DTYMK* and a neurodegenerative condition [17]. In the current study, functional evidence and clues towards the pathophysiological mechanism are provided for the first time. We describe two individuals with pathogenic bi-allelic variants in *DTYMK* and detail the clinical and biochemical effects of these deleterious variants in the individuals and *dtymk*-deficient zebrafish.

## Materials and methods

### Clinical samples, materials and exome sequencing

DNA samples and skin biopsies were obtained from subjects in this study following written informed consent in a clinical genetic diagnostic setting. DNA was isolated from peripheral blood and fibroblasts were grown from skin biopsies. Photographs of affected individuals, used in this study were obtained after written approval from their legal guardians. Dermal fibroblasts were grown from punch skin biopsies in Minimal Essential Medium supplemented with l-glutamine, antibiotics (Pen-Strep) and 15% fetal bovine serum at 37 °C in a humidified atmosphere containing 5% CO_2_.

Exomes were captured using the SureSelectXT Human All Exon 50 Mb kit (Agilent Technologies) and sequenced on an Illumina HiSeq2000 by BGI-Europe (BGI Europe). Read mapping was performed using BWA (Burrows-Wheeler Aligner) and variant calling using GATK (Genome Analysis ToolKit).

### Brain histology and immunohistochemistry

The brain was fixed in buffered 4% formalin for 3 weeks and grossly sectioned. Tissue blocks were embedded in paraffin and cut at 5 µm thickness. Tissue sections were stained with Hematoxylin and Eosin (H&E), Klüver-periodic acid Schiff (Klüver-PAS) and Nissl according to routine methods. Immunohistochemistry was carried out as described [14] with antibodies against the astrocyte protein glial fibrillary acidic protein (GFAP; Millipore, 1:1000), the endothelium marker CD34 (DAKO, 1:200), the microglia marker Iba-1 (Wako, 1:10.000), the major mature myelin protein proteolipid protein (PLP; Serotec, 1:3000) and the apoptosis marker caspase3 (DAKO, 1:500). Positive and negative controls by omitting the primary antibody were included in each experiment. Briefly, sections were deparaffinized and rehydrated. Endogenous peroxidase activity was quenched by incubating the slides in 0.3% hydrogen peroxide in methanol. Slides were rinsed with distilled water and transferred to citric acid (pH6). Heat-induced antigen retrieval was performed using microwave irradiation for 15 min on low setting. Tissue sections were then cooled to room temperature and incubated overnight with primary antibodies. The antibody binding was visualized with 3, 3’-diaminobenzidine (DAB) staining. Sections were then counterstained with hematoxylin, mounted with polyvinyl alcohol medium with Dabco (Sigma) and photographed using a Leica DM6000B microscope (Leica Microsystems).

### Thymidylate kinase (dTMPK) assay

In vitro dTMPK activity was determined as previously described [37]. Briefly, samples were thawed on ice in buffer A (320 mM sucrose, 5 mM Tris pH 7.4, 2 mM EGTA (ethylene glycol-bis(β-aminoethyl ether)-N,N,N′,N′-tetraacetic acid), Triton X-100, 0.05% and protease inhibitors) and homogenized. Subsequently, homogenates were centrifuged at 16 000×*g* for 15 min at 4 °C to pellet cellular debris. dTMPK activity was measured by incubating the protein extracts in a reaction mixture containing 50 mM Tris/HCl, pH 7.6, 0.5 mg/ml bovine serum albumin, 5 mM MgCl_2_, 5 mM Dithiothreitol, 5 mM ATP, 30 mM Sodium fluoride (NaF), 0.1% Triton X-100 and 25 µM tritium-labeled dTMP ([^3^H]-dTMP), which was prepared from [^3^H]-thymidine using thymidine kinase catalyzed reaction. At 0, 10, 20 and 30 min., 10 µl aliquots were removed and spotted onto Diethylaminoethyl cellulose (DEAE) filter paper (PerkinElmer) and dried. The filter papers were then washed in 50 mM ammonium formate and sorted into scintillation vials. Reaction products were eluted with 0.5 ml of 0.2 M KCl and 0.1 M HCl, mixed with scintillation fluid and countered in a liquid scintillation counter (Tricarb, PerkinElmer).

### Zebrafish husbandry and genome editing

Zebrafish (*Danio rerio*), AB strain, were housed in recirculating systems on a 14/10 day–night regime [18]. All animal studies were performed in concordance with the European and local guidelines and regulations of Maastricht University on animal experimentation.

Care of animals and animal experiments were conducted exclusively by licensed staff.

CrispR/Cas9-mediated genome editing was performed as described [10, 33]. A guideRNA (gRNA) targeting exon 4 (GGGACTTCCAAAACCAGACC) was designed using the CHOPCHOP tool [16]. The gRNA was in vitro translated using the MEGAShortscript T7-kit (ThermoFisher Scientific). Fertilized oocytes were injected with 100 ng/μl gRNA and 200 ng/μl Cas9 mRNA (in vitro transcribed with the SP6 mMESSAGE mMACHINE kit (ThermoFisher Scientific) using the pCS2-nCas9n plasmid as a template) [10]. Genetically modified founders were identified by genotyping embryos from outcrosses using HRM (High-Resolution Melting curve analysis; see genotyping section) with primers HRMtmpk_exon4For and HRMtmpk_exon4Rev (Supplementary Table 1, online resource). Modification of the locus was confirmed using Sanger sequencing.

### Genotyping

Sanger sequencing was performed on lysates of caudal fin biopsies of adult zebrafish, embryo lysates or purified DNA from human blood or fibroblasts. To this end, target regions were amplified by PCR and sequence analysis in both directions was performed using the ABI Big Dye Terminator Cycle Sequencing Ready Reaction kit and the ABI3730 Genetic Analyzer (Applied Biosystems). The primers used to genotype the human samples were: DTYMK_05_F and DTYMK_05_R. Genomic modification of the zebrafish *dtymk* locus was confirmed with M13-labeled primers zfdtymk_ex4For and zfdtymk_ex4Rev (Supplementary Table 1, online resource).

For high-resolution melting curve (HRM) analysis, zebrafish embryos were euthanized with a lethal dose of MS222 (tricaine). Biopsies from the caudal fin of adult fish were taken after anesthesia with MS222. Embryos or fin clips were placed individually in lysis buffer, consisting of 1 M KCl, 1 M MgCl_2_, 1 M Tris pH 8.3, Nonidet P40, Tween-20 and gelatine, supplemented with Proteinase K (100 µg/ml). Lysis was performed for 1 h at 60 °C, 15 min at 95 °C and then held at 4 °C. After adding 80 µl of water, lysates were used for PCR. Amplification and HRM analysis were performed in the LightCycler ®480 (Roche Applied Science), using the HRM Master kit (Roche Applied Science) according to the following conditions: 95 °C for 10 min; 45 cycles of 95 °C for 10 s, 59 °C for 15 s, 72 °C for 15 s; one cycle of 95 °C for 60 s, 40 °C for 60 s, and a melting from 60 °C to 95 °C rising at 0.02 °C per second. All samples were run in triplicate. HRM analysis was performed using the software LightCycler ® 480 Gene Scanning version 1.5.1.

### Whole-mount antibody staining and histology

Zebrafish embryos were fixed in 4% paraformaldehyde and dehydrated to 100% methanol and stored at − 20 °C until further processing. Prior to antibody staining, embryos were rehydrated to PBT (PBS + 0.1% Tween) and permeabilized using 10 μg/ml proteinase K. After refixation in 4% paraformaldehyde, blocking was performed with 10% lamb serum, 0.1% Dimethyl sulfoxide (DMSO) for at least 3 h at room temperature. Next, the primary antibody was added for at least 2 h. After extensive washing, the secondary antibody was added for at least 2 h. 3, 3ʹ-diaminobenzidine (DAB)-staining was performed using the liquid DAB + substrate chromogen system (Dako). Stained embryos were visualized and photographed using a stereomicroscope (Leica M165 FC). The antibodies used in this study were: anti-γH2AX (gamma H2A histone family member X) (GeneTex GTX127342) and anti-phospho-Histone H3 (Ser10) (pH3) (Millipore 06-570).

For histological analysis, embryos were fixed in 4% paraformaldehyde and washed in phosphate buffered saline (PBS). Next, they were embedded in 2% low-melting point agarose to enable correct orientation for sectioning. Agarose blocks were trimmed, dehydrated and embedded in paraffin. 5 μm sections were cut, air dried and stained with eosin/hematoxylin. Sections were analyzed and photographed on a Nikon eclipse e800 microscope.

Apoptotic cells in zebrafish embryos were visualized using TUNEL (Terminal deoxynucleotidyl transferase dUTP nick end labeling) staining by means of the in Situ Cell Death Detection Kit (Roche) according to the manufacturers’ instructions.

### Head size in larval zebrafish

The size of the head was estimated by measuring the area between the eyes of embryos at 2 day post-fertilization (dpf) as described in Stankiewics et al*. *[32]. Briefly, pictures of the dorsal view of embryos were taken and the area of the region between the eyes was quantified using ImageJ software.

### DNA damage response signalling

To assess the DNA damage response in vivo, zebrafish embryos at 24 hpf (hours post-fertilization) were UV-irradiated in a UV Stratalinker 1800 (Stratagene) at a dose of 12,000 microjoule/cm^2^. Subsequently, they were left to recover for 24 h at 28 °C and processed for antibody staining with the anti-γH2AX antibody.

### EdU labeling

Fibroblasts were labelled with 10 μM EDU (5-ethynyl-2'-deoxyuridine) for 30 min, then processed using Click-IT technology as described by the manufacturer (ThermoFisher Scientific). Subsequently, cells were analyzed in a BD Accuri C6 Plus flow cytometer. Profiles were processed using BD CSampler software (BD Biosciences).

### Alkaline gel electrophoresis

Incorporation of ribonucleotides was investigated using alkaline agarose gel electrophoresis as described in Sambrook et al*.* [31]. Genomic DNA was isolated using Qiagen Blood and Tissue kit and incubated at 55 °C for 2 h in the presence of 300 mM NaOH before electrophoresis on alkaline agarose gels. DNA was visualized by ethidium bromide staining or SYBR Gold staining (Thermo Fisher).

### Mitochondrial measurements

Mitochondrial DNA (mtDNA) copy number was measured in genomic DNA preparations. mtDNA copy number was assessed by comparing expression levels of a mitochondrial encoded gene (*ND1*) versus a nuclear encoded gene (*B2M*). Relative mtDNA copy number was calculated by calculating ΔCT (= CT nuclear gene, B2M–CT mitochondrial gene, ND1) and converting ΔCT to relative expression levels (relative expression = 2^∆*CT*^) as described in Rahn et al. [27].

The integrity of the mitochondrial genome was assessed by PCR amplification of the complete mitochondrial genome by long-range PCR using Phusion Hot Start polymerase II using primers MM_16426_NGS_F and MM16425_NGS_R (Supplementary Table 1, online resource), followed by agarose gel electrophoresis.

Complex activity in fibroblasts of individual I and parents was analyzed as follows: fibroblasts were cultured and harvested for enzyme analysis. Complex activities were determined as described by Rodenburg and coworkers [29]. This assay is a diagnostic assay, performed at the Translational Metabolic Laboratory of the Radboud UMC.

### Statistical analysis

Statistical evaluation and data presentation were performed using GraphPad Prism 8 software. Microsoft Excel was used to calculate the exact *p* values. Statistical significance was calculated using the Student’s *t* test (unpaired, two-sided). Differences were considered statistically different when *p* < 0.05. Variance is calculated when comparing different groups and was equal unless indicated otherwise.

## Results

1. Clinical phenotypes

### Individual I

The proband was the first child of unrelated Dutch parents (Fig. 1b). Family history was uneventful with no prior miscarriages. Pregnancy was induced by in vitro fertilization because of male subfertility and was complicated by polyhydramnios. Third-line ultrasound examinations at 30th and 36th weeks of gestation, however, showed no explanation for the polyhydramnios. No congenital abnormalities were observed. Femur length growth was at the 10th centile. The fetal brain looked normal and the fetus had a normal fetal head circumference, also at the 10th centile (Table 1). There was no known teratogenic exposure.

Delivery was uncomplicated at 37 weeks of gestation. Birthweight was 2645 g (15th centile Fenton growth chart for girls 37 weeks of gestation; 25th centile Perined), occipitofrontal circumference (OFC) was 31 cm (2nd centile) and height was 44 cm (10th centile); Apgar scores were 9/10 after 1 and 5 min, respectively. No apparent dysmorphic features were observed (Table 1). Because of poor feeding, she was readmitted to the hospital on the 3rd day. Nutrition with naso-gastric tube was needed and well tolerated. However, normal growth was never achieved. The OFC and height progressively deviated from the normal centiles. She developed severe microcephaly with a very puffy appearance (Fig. 1b). The girl was hypotonic at birth but developed spasticity with opisthotonus within 1 year of age. No neurodevelopmental milestones were achieved. No eye contact was ever made. She developed recurrent febrile seizures with flat trace EEG at 6 months of age which were successfully treated with phenobarbital.

Brain MRI at age 3 weeks of life showed slight underdevelopment of the caudate nucleus and the frontal cortex, with severe underdevelopment of the putamen. Brain MRI at 6 months of age showed dramatic atrophy of the cerebral hemispheres with severe enlargement of the lateral ventricles and subarachnoid spaces (Fig. 1b, individual I). At the basal ganglia, only the thalamus appeared of normal volume. On MRI, the brain stem and cerebellum also appeared normal (Fig. 1b, individual I). Microarray showed a normal karyotype: 46, XX.

Extensive metabolic investigations were performed including urinary organic acids, amino acids, purines & pyrimidines, glycosaminoglycans, oligosaccharides, acylglycines, creatine and guanidinoacetate, bile acids and bile alcohols. Plasma amino acids, acylcarnitines, very-long chain fatty acids, sterols, chitotriosidase, creatine and guanidinoacetate, sterols, and plasmalogens were normal. CSF amino acids were without abnormalities. At 18 months of age she had a respiratory illness. This was complicated by cardio-pulmonary arrest, leading to demise.

**Fig. 2:**
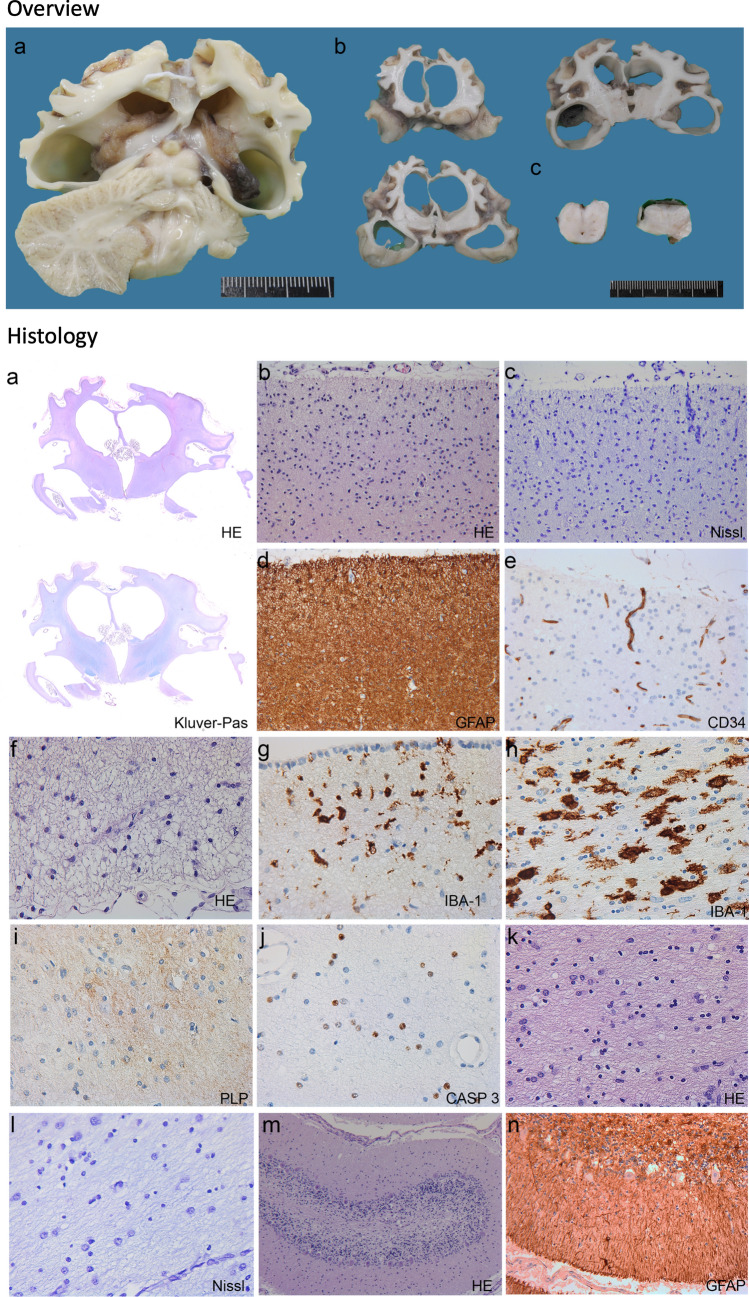
Neuropathology. Overview (upper) Panel. Macroscopic aspect of the brain of individual I. (**a**, **b**) Coronal cut through the cerebral hemispheres (**a**, **b**), cerebellum (**a**) and brainstem (**c**, pons on the left, medulla oblongata on the right). Histology (lower) panel. Microscopic aspect of the brain of individual I. **a** Whole mounts of the cerebral hemispheres confirm the massive degree of global atrophy (**a**, haematoxylin and eosin, HE) and show paucity of myelin throughout the cerebral hemispheres and capsules (Kluver-PAS). **b**–**e** Photomicrographs of the cerebral cortex from the frontal lobe show loss of neurons, including pyramidal cells (**b**, HE; **c**, Nissl stain for nuclei), astrogliosis (**d**, glial fibrillary acidic protein, GFAP) and minimal degree of vascular proliferation (**e**, CD34). **f**, **g** Photomicrographs of the deep white matter adjacent to the lateral ventricles show loss of oligodendrocytes (**f**, HE) and moderate activation of microglia (IBA-1). **h**–**j** The more peripheral hemispheric white matter shows massive activation of microglia with ameboid morphology (**h**, IBA-1), lack of myelin (**i**, proteolipid protein stain for myelin) and loss of oligodendrocytes by apoptosis (**j**, CASP3 stain for apoptotic cells). **k**, **l** Photomicrographs of the basal ganglia (**k**, HE) and thalamus (**l**, Nissl) show loss of neurons and reactive gliosis. **m**, **n** In the cerebellum, microscopic examination shows loss of neurons in the granular and Purkinje cell layer (**m**, HE) and moderate reaction of the Bergmann glia (**n**, GFAP)

Post-mortem examination of the internal organs showed no abnormalities. Gross neuropathological examination showed severe atrophy of the neocortex, cerebral white matter and basal ganglia, with normal size of brainstem and cerebellum (Fig. 2 Overview). Microscopy of the cerebral cortical ribbon revealed massive loss of neurons with astrogliosis and minimal vascular proliferation (Fig. 2 Histology). Whole mounts of the cerebral hemispheres at the level of the neostriatum confirm the massive degree of global atrophy (Fig. 2a, haematoxylin & eosin, HE) and show paucity of myelin throughout the cerebral hemispheres and capsules (Fig. 2a, Kluver-PAS). Photomicrographs of the cerebral cortex from the frontal lobe show loss of neurons, including pyramidal cells (Fig. 2b, HE; c, Nissl stain for nuclei), intense anisomorphic astrogliosis (Fig. 2d) and minimal degree of vascular proliferation (Fig. 2e). Photomicrographs of the deep white matter adjacent to the lateral ventricles show loss of oligodendrocytes (Fig. 2f, HE) and moderate activation of microglia (Fig. 2g). The more peripheral hemispheric white matter shows massive activation of microglia with ameboid morphology (Fig. 2h), lack of myelin (Fig. 2i) and loss of oligodendrocytes by apoptosis (Fig. 2j). Photomicrographs of the neostriatum (Fig. 2k, HE) and thalamus (Fig. 2l, Nissl) show loss of neurons and reactive gliosis. In the cerebellum (Fig. 2m, n), microscopic examination shows loss of neurons in the granular and Purkinje cell layer (Fig. 2m, HE) and moderate reaction of the Bergmann glia (Fig. 2n, GFAP).

### Individual II

This boy was the second child of healthy consanguineous Egyptian parents (first cousins, Fig. 1b). There is a healthy older sister. Pregnancy was uneventful, with no known teratogenic exposure. At the 32nd week of gestation, the prenatal ultrasound showed smaller biparietal diameter for gestational age. The individual was born at term by elective cesarean. His birth weight was 2250 g (− 1.8 SD) (Table 1). His birth height and OFC were not documented but the latter was noticed to be small. He stayed in the neonatal intensive care unit for 7 days because of neonatal jaundice that was treated with phototherapy. The individuals’ parents became concerned during the first months of life as he did not offer eye contact or track objects. At the age of 7 months, he presented with fever and repeated vomiting and was admitted to the hospital. Severe anemia was detected and treated by blood transfusion. The individual underwent multiple analyses of hematological indices and liver enzymes at different occasions that always showed microcytic hypochromic anemia and elevated liver enzymes, respectively.

He developed severe myoclonic jerks at 15 months of age, which were treated with carbamazepine. The EEG demonstrated slow background activity and occasional sharp waves. Metabolic work-up including glucose, lactate, ammonia, biotinidase, creatine kinase, acylcarnitines, amino acids, very long-chain fatty acids and urinary organic acids profile were all normal.

At 30 months of age, the boy was referred for genetic counseling. On examination, his weight was 7200 g (− 4 SD), height was 68 cm (− 5.4 SD), and OFC was 38 cm (− 7.4 SD) (Table 1). He had fairer hair and skin compared to his parents and sister. Bitemporal narrowing, full cheeks, puffy hands and feet were noted (Fig. 1b, individual II). Furthermore, he had bilateral undescended testes and micropenis. There was good control of the head and increased tone distally in the arms and legs. Tendon reflexes were increased, with bilateral clonus and positive Babiniski signs. He made almost no developmental progress: he did not roll over, sit, vocalize, or smile. Ophthalmologic examination and hearing test were normal. Serum electrolytes, urea, creatine, albumin level and 17-hydroxyprogesterone were all normal. Chromosome analysis showed a normal 46, XY karyotype. Brain MRI and CT at age 2 years showed profound generalized cerebral atrophy with widening of the subarachnoid spaces (Fig. 1b, individual II). The basal nuclei were small, but visible, while the thalamus appeared to have a normal size. Also, the brain stem and cerebellum were spared (Fig. 1b, individual II). At the age of 32 months, the boy developed a pneumonia followed by coma and death.

At birth, no apparent dysmorphic features were observed in both children. In the months following birth, the following phenotypic features became apparent in both individuals: growth retardation, puffy body, seizures, failure to reach developmental milestones, microcephaly and severe, progressive atrophy of the cerebral hemispheres and basal ganglia (Table 1). Pathology in individual I confirms massive neuronal dropout, only sparing the dentate nucleus and brain stem.

2. Description of variants in *DTYMK*

Exome sequencing identified compound heterozygous variants in *DTYMK* (Deoxy Thymidylate Kinase) in family I: Chr2(GRCh37):g242619732G>A; NM_012145.3:c.242C>T; p.Pro81Leu (paternal) and Chr2(GRCh37):g242618013C>T; NM_012145.3:c.382G>A; p.Asp128Asn (maternal) (Fig. 1b; Table 1). In the second case (individual II), one of the abovementioned variants, Chr2(GRCh37):g242619732G>A;NM_012145.3:c.242C>T; p.Pro81Leu was observed in a homozygous state. (Fig. 1b; Table 1).

Variant c242C>T, p.(Pro81Leu) is a missense substitution of a highly conserved nucleotide (phyloP: 5.29 [− 14.1; 6.4]) leading to a change in a moderately conserved amino acid (considering 13 species) (Fig. 1d). Predictions of the amino acid change, p.(Pro81Leu), varied from tolerated (SIFT, score: 0.19, median: 2.82) to “possibly damaging” (PolyPhen2 score 0.885) to disease causing (MutationTaster, *p* value: 1). This variant is unknown to ESP and ExAC. In dbSNP, it is known as rs1267106442 and is found once in gnomAD with a MAF of 0.0000319, but only in a heterozygous state (accessed on 04/05/2021) (Supplementary Fig. 2a, online resource).

The second variant, c.382G>A, p.(Asp128Asn) is also a missense substitution involving a highly conserved nucleotide (phyloP: 5.61 [− 14.1; 6.4]), giving rise to a change in a highly conserved amino acid, up to *Saccharomyces cerevisiae* (considering 13 species) (Fig. 1c, d). SIFT-prediction was tolerated (score: 0.09, median: 2.82), whereas MutationTaster and Polyphen-2 predicted this variant to be disease-causing (*p* value: 1) and probably damaging (score 1.00), respectively (Supplementary Fig. 2a, online resource). This variant is known to bSNP as rs373875797 (MAF/MinorAlleleCount: T = 0.000/0) and to ESP as ESP6500SIV2. This variant has been reported twice in the ExAC database (MAF: 0.00001649), but never in a homozygous state. This variant is known to gnomAD and appears 4 times with a MAF of 0.00001591, but only in a heterozygous state (accessed on 04/05/2021). Both variants are not located in a known functional domain of the protein (Fig. 1c).

3. Zebrafish *dtymk* knockout model

**Fig. 3:**
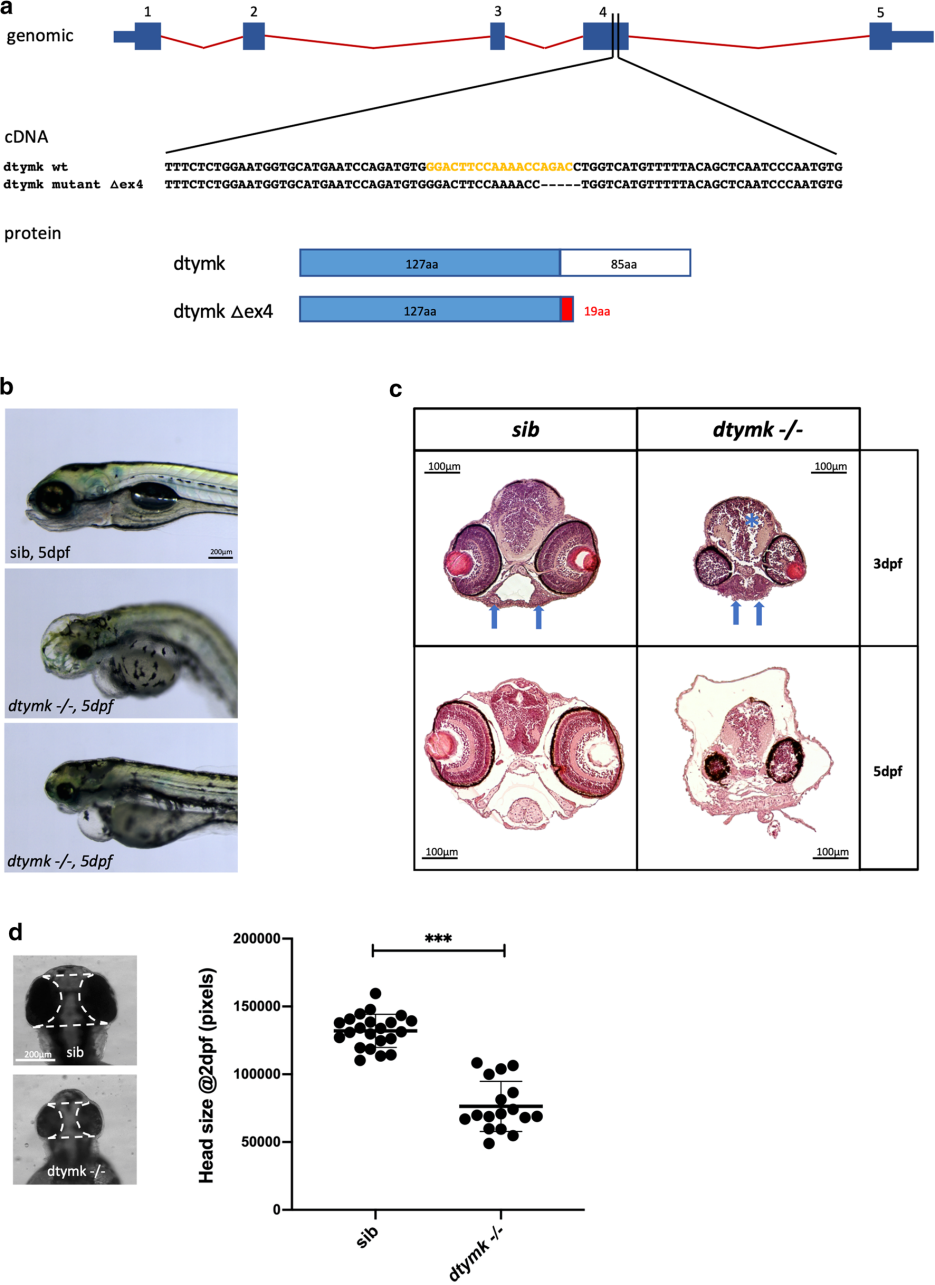
*dtymk* knockout zebrafish. **a** Genomic location of the gRNA sequence used to generate the knockout line of *dtymk,* indicated in yellow. The mutant line, *Δex4* contains a 5-bp deletion in exon 4 resulting in a truncated protein. **b** Phenotype of *dtymk* knockout (*dtymk –/–*) embryos at 5dpf and their siblings (sib). The homozygous mutant embryos (*dtymk –/–*) show small, irregular eyes and edema around the brain, heart and intestine. The scale bar represents 200 μm. **c** Hematoxylin/eosin staining of transversal sections through the head of sibling and *dtymk* mutant embryos at 3dpf and 5dpf at the level, where the optic nerve exits the eye. Images show prominent degeneration of brain tissue (blue star) and structural abnormalities of the eyes and jaw cartilage structures (blue arrow) in the mutant embryos (*dtymk –/–*) which is absent in the sibling (sib) embryos. The scale bar represents 100 μm. **d** Quantification of head size in *dtymk* –/– and sibling (sib) embryos at 2dpf. Average head size of 19–24 embryos at 2dpf showing significant microcephaly in mutant vs. sibling embryos (***, *p* = 1.52 × 10^–13^). Error bars show the standard deviation. Two biological replicates. The scale bar represents 200 μm

To study the relationship between neurodegeneration and dTMPK deficiency, we generated a *dtymk* knockout model in zebrafish (Fig. 3). Sequencing of homozygous mutant embryos revealed a 5-bp deletion in exon 4 (Fig. 3a). This modification leads to a premature stop codon, 19 amino acids downstream of the deletion, whereas the wildtype protein contains another 85 amino acids. The first 127 amino acids (60%) of the tmpk protein upstream remain unaltered (Fig. 3a).

At 5 dpf, all homozygous *dtymk* mutant embryos show small eyes, pericardial edema and edema around the intestine, while 2/3 of the mutant embryos also had extensive edema of the brain (Fig. 3b; Supplementary Fig. 2b, online resource). The fraction of affected embryos was consistent with the expected fraction from a heterozygous incross (25%), and confirmed by genotyping (Supplementary Fig. 2b, online resource). From 3dpf onwards, *dtymk* mutant embryos can be morphologically distinguished from their siblings as the eyes are markedly smaller and pericardiac edema is prominent. Remarkably, mutant embryos at 3dpf show twitching movements, reminiscent of epileptic seizures although other causes (eg. motor neuron dysfunction) cannot be excluded. From 4dpf onwards, mutant embryos develop prominent edema of the brain and around the intestine. At 5dpf, more than 40% of mutant embryos have died. Since affected individuals show microcephaly, the head size of mutant and sibling embryos was quantified at 2dpf (Fig. 3d). A timepoint early in development was chosen to avoid brain edema interfering with the assessment of head size. Head size was found to be significantly (*p* = 1.52 × 10^–13^) smaller when compared to sibling embryos of the same batch (Fig. 3d). The length of sibling vs. mutant embryos was not different (Supplementary Fig. 1c), ruling out developmental delay as the cause of microcephaly in *dtymk* mutant zebrafish.

Histology of 3- and 5-day-old embryos provided a more detailed insight into the phenotype (Fig. 3c). In the brain, empty spaces, indicative of neurodegeneration are apparent in the mutant embryos (Fig. 3c, blue star). In addition, cartilage structures of the lower jaw (Meckels’ cartilage) are absent in the mutant larvae (Fig. 3c blue arrows). Severe underdevelopment of the eyes is prominent: the eyes of mutant embryos are smaller, have an irregular shape and only show the retinal pigmented epithelium (RPE) and photoreceptor layer (PRL), whereas the other 4 layers are absent (Supplementary Fig. 2d, online resource).

**Fig. 4:**
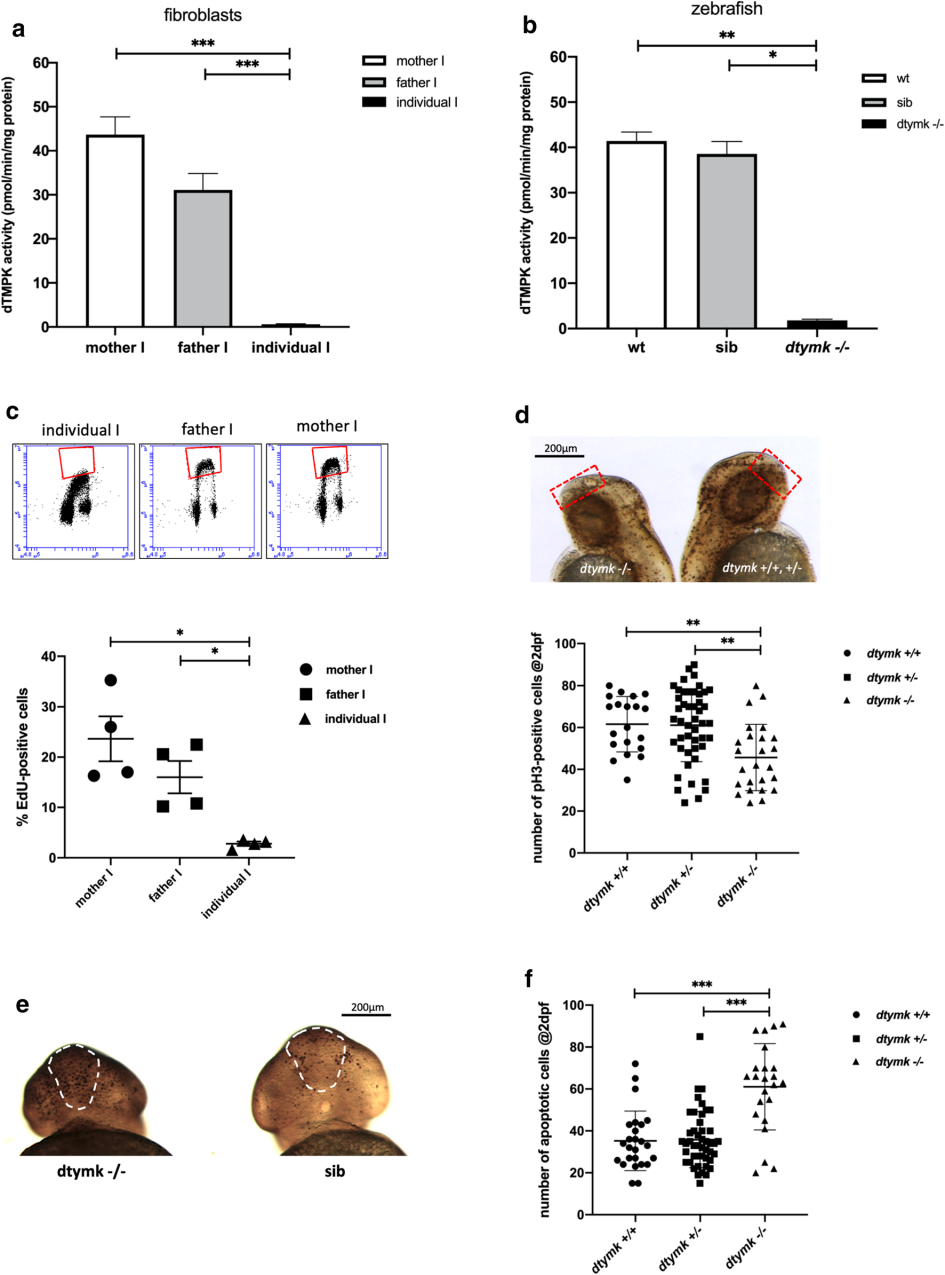
dTMPK activity and replication defects in fibroblasts and zebrafish. **a** dTMPK activity measured in fibroblasts of family I. In both parents, activity was readily detected and was lower in the fibroblasts of the father. dTMPK activity was hardly detectable in individual I (*n* = 6). Mother vs. individual I (***, *p* = 1.46 × 10^–6^, unequal variance). Father vs. individual I (***, p = 5.77 × 10^–6^, unequal variance). **b** dtmpk activity in lysates of zebrafish at 5dpf. Lysates were prepared from pools of 35–50 embryos of indicated genotypes. *n* = 2–3 and measurements were performed 2 times. dtmpk activity was negligible in mutant embryos and robustly detected in both sibling embryos as embryos of the same age from an unrelated wildtype strain (AB). Wt vs. mut: **, *p* = 0.0001); Sib vs. mut: *, *p* = 0.0028. **c** EdU incorporation in the fibroblasts of family I. Representative flow cytometry profiles are shown from fibroblasts of the individual and the parents. The red boxes denote the EdU positive (S-phase) cell populations. Quantification of the percentage of EdU-positive (S-phase) cells shows that the fibroblasts of individual I have significantly fewer proliferating cells when compared to the fibroblasts of the mother (*, *p* = 0.0180, unequal variance) and father (*, *p* = 0.0249, unequal variance). Indicated values are average % of EdU-positive cells ± SEM, *n* = 4). **d** Whole-mount pH3-immunostaining showing proliferating cells in wildtype (*dtymk* + / +), heterozygous (*dtymk*+/−) and *dtymk*-knockout (*dtymk* –/–) embryos at 2dpf. Representative images of side-view of an embryo with many proliferating cells (black dots, right image) and an embryo with less proliferating cells (left image). The red, dotted rectangle represents the area that was analyzed for the quantification of the number of proliferating cells in the forebrain. The scale bar represents 200 μm. Quantification of the number of pH3-positive cells in the brain indicates that the mutant embryos have significantly less pH3-positive cells in the brain when compared to wildtype (**, *p* = 0.0008) and heterozygous (**, *p* = 0.0004) embryos, *n* = 9–24 embryos per group, two biological replicates. Indicated values are average ± SEM. **e** Whole-mount TUNEL staining showing apoptotic cells in sibling (sib: *dtymk*+/+or *dtymk*+/−) and *dtymk*-knockout (*dtymk* –/–) embryos at 2dpf. Representative frontal images of an embryo with many apoptotic cells (black dots, left image, *dtymk –/–*) and an embryo with less apoptotic cells (right image, sib). The white, dashed area represents the forebrain, the area that was analyzed for the quantification of the number of apoptotic cells in the forebrain. Embryos were genotyped after staining and imaging. The scale bar represents 200 μm. **f** Quantification of the number of apoptotic cells in the forebrain in wildtype, heterozygous and *dtymk*-knockout embryos at 2dpf. The mutant embryos have significantly more (***, *p* < 0.0001) apoptotic cells in the forebrain when compared to wildtype (***, *p* = 6.45 × 10^–6^, equal variance) and heterozygous (***, *p* = 7.54 × 10^–6^, unequal variance) embryos, *n* = 23–46 embryos per group, two biological replicates. Indicated values are average ± standard deviation

TUNEL staining at 2dpf revealed higher amounts of apoptotic cells in *dtymk* mutant zebrafish. We quantified the number of TUNEL-positive cells in the forebrain of *dtymk* mutant zebrafish and found that dtymk mutant embryos have significantly more apoptotic cells in the forebrain when compared to wildtype (*p* = 6.45 × 10^–6^) or heterozygous (*p* = 7.54 × 10^–6^) siblings (Fig. 4e, f).

These results, together with the observation of extensive postnatal cerebral atrophy in both individuals and the presence of apoptotic oligodendrocytes in the brain of individual I (Fig. 2j) demonstrate that neuronal cell death is a hallmark of the disease phenotype as it is prominent in both the affected individual as the zebrafish model as a result of dtymk deficiency.

4. *DTYMK* alleles in affected individuals and zebrafish are loss-of-function

The functional effect of the variants, observed in the individuals and the deletion in the zebrafish model were assessed at the biochemical level using an in vitro dTMPK assay [37]. To this end, dTMPK activity was measured in cultured fibroblasts of family I (Fig. 4a). In fibroblasts of the parents, dTMPK activity was readily detectable. In the mother, an activity of 43.65 pmol/min/mg protein was measured, whereas the cells of the father showed lower activity: 31.08 pmol/min/mg protein. dTMPK activity of the individual was hardly detectable: 0.62 pmol/min/mg protein. dTMPK enzyme activity of the individual is significantly lower than those of the parents (mother vs. individual I *p* = 1.46 × 10^–6^; father vs. individual *p* = 5.77 × 10^–6^).

In the zebrafish model, pools of 35–50 larvae of 5dpf were phenotyped and processed for dTMPK enzyme activity measurements (Fig. 4b). The sibling embryos had a dtmpk activity of 38.55 pmol/min/mg protein, which is comparable to the activity measured in the fibroblasts of the parents. *Dtymk* mutant embryos on the other hand had barely detectable activity: 1.80 pmol/min/mg protein. Unrelated wildtype larvae of the same age showed a dtmpk activity of 41.43 pmol/min/mg protein. Dtmpk activity of mutant larvae is significantly lower than those of sibling larvae (*p* = 0.0028) or wildtype larvae (*p* = 0.0001) (Fig. 4b).

Similar results from fibroblasts of an affected individual and knockout zebrafish confirm that the tested alleles in fibroblast and zebrafish represent loss-of-function alleles, giving rise to a non-functional dTMPK enzyme, shown by the very low dTMPK activity in both models.

5. *DTYMK* disease alleles cause impaired DNA replication, elevated ribonucleotide incorporation and altered DNA damage signaling

Since the effect of the variants in *DTYMK* completely blocks the known biosynthesis pathway of dTTP, we evaluated processes that require significant amounts of nucleotides including DNA replication and aspects of DNA repair. To assess DNA replication, we performed pulse-EdU staining of fibroblasts from individual I and the parents to quantify the number of cells in S-phase of the cell cycle (Fig. 4c). Flow cytometry analysis of EdU-labeled fibroblasts showed that in the parents of family I, the number of S-phase cells is 23.65% and 16.03% in the mother and father, respectively. In contrast, the fraction of S-phase cells in individual I fibroblasts was only 2.8%. This is significantly lower than the amounts in the fibroblasts of the mother (*p* = 0.0180) and father (*p* = 0.0249) (Fig. 4c). These results show that DNA replication is impaired in fibroblasts of affected individuals.

As the principal phenotype in the individuals is neurological, we next assessed cell proliferation in the brain of *dtymk* mutant zebrafish larvae at 2dpf using phospho-Ser10 Histone H3 (pH3) staining. Histone H3 is phosphorylated at Ser10 by cyclin dependent kinase 1 in late G2 phase and mitosis and is a frequently used marker for cell proliferation. The number of proliferating cells in the forebrain of *dtymk* mutant and sibling embryos at 2dpf was evaluated (Fig. 4d). Quantification was limited to the forebrain as this is the homologous structure to the human cerebral cortex, which is most affected in the affected individual. As the larvae are morphologically undistinguishable at 2dpf, the number of proliferating cells in the forebrain of individual embryos was counted and embryos were subsequently genotyped. We found the number of pH3-positive cells in the forebrain of *dtymk* mutant embryos to be significantly lower than the number of proliferating cells in wildtype (*p* = 0.0008) and heterozygous (*p* = 0.0004) *dtymk* embryos (Fig. 4d). When comparing the sibling embryos, there is no difference in the number of proliferating cells in wildtype versus heterozygous *dtymk* embryos. In summary, we find severe defects in DNA replication in individual I-derived fibroblasts and in proliferation in the brain of *dtymk* mutant zebrafish.

**Fig. 5:**
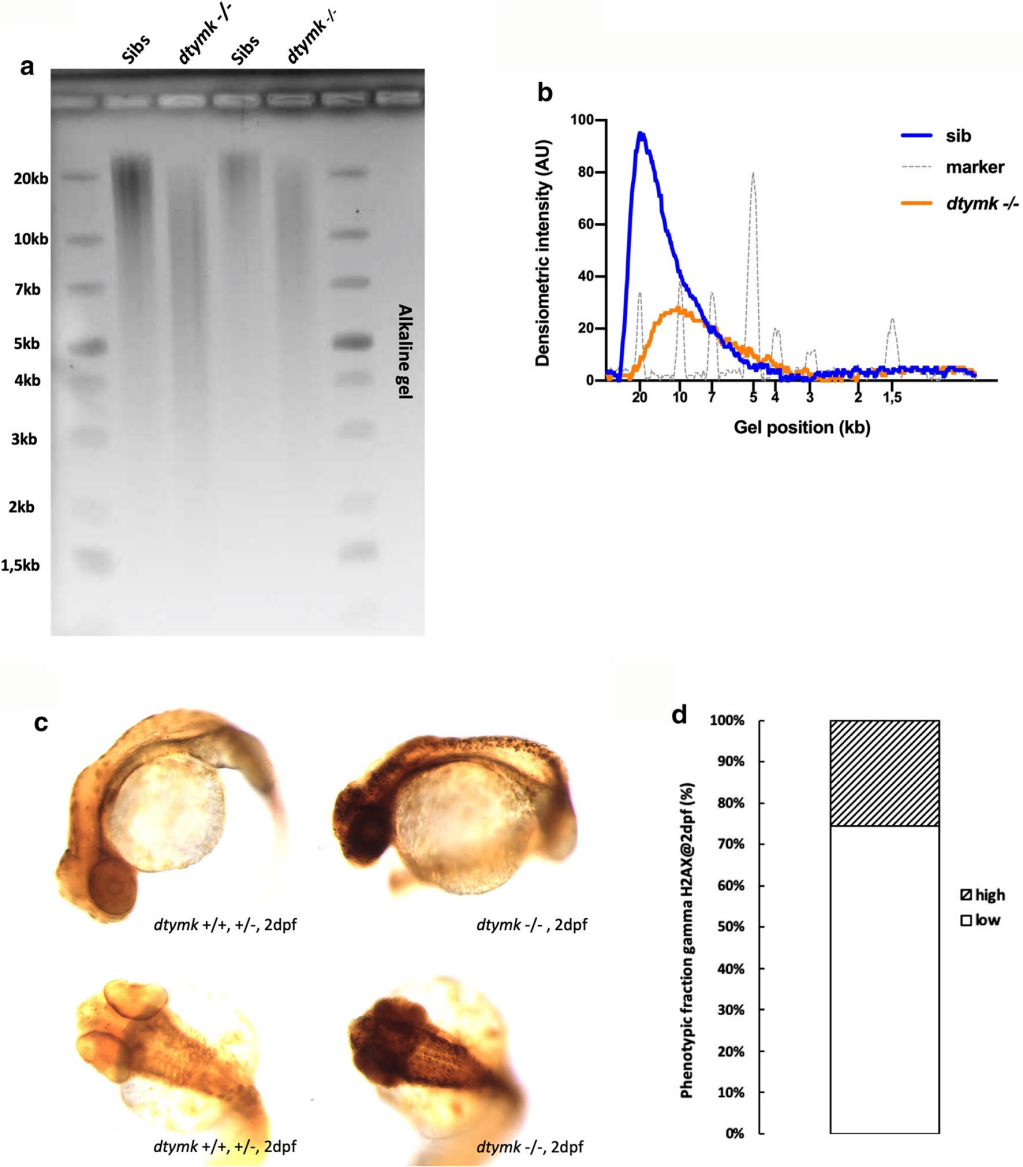
Ribonucleotide incorporation and DNA damage signaling. **a** Representative image of alkaline gel electrophoresis of genomic DNA. Genomic DNA of dtymk mutant embryos (*dtymk –/–*) and sibling (sibs) embryos at 5dpf was subjected to alkaline gel electrophoresis. The first two lanes contain 5 μg of genomic DNA of sibling (Sibs) and *dtymk* homozygous mutant (*dtymk –/–*) embryos, respectively. The following two lanes contain 2.5 μg of genomic DNA of sibling (Sibs) and *dtymk* homozygous mutant (*dtymk –/–*) embryos, respectively. The first and last lane contain the marker Generuler 1 kb Plus DNA ladder (Thermo Fisher). Three biological replicates produced highly similar results. **b** Quantification of alkaline gel electrophoresis. Densiometric intensity values of a line drawn from the slot to the bottom of the gel were determined using ImageJ and plotted as a function of distance in the gel. The genomic DNA of homozygous mutant (*dtymk –/–*) embryos, marked in orange, migrates at a lower molecular weight as the DNA of sibling embryos, marked in blue. Results indicate an increased sensitivity to alkaline hydrolysis of the genomic DNA of homozygous *dtymk* mutant (*dtymk –/–*) embryos. The position of the molecular weight marker is indicated by the grey line and its respective peaks corresponding to different molecular weights are indicated in the *x*-axis (gel position). **c** Representative images of side view (upper panels) and dorsal view (lower panels) of embryos at 2dpf showing sites of DNA-damage, visualized by γH2AX-staining (black dots). DNA damage was induced by UV-irradiation at 24hpf followed by a recovery time of 24 h. The left panels show sibling embryos (*dtymk*+/+, +/−) and the right panels show mutant (*dtymk* –/–) embryos. The mutant embryos clearly show many sites of unrepaired DNA-damage, whereas the sibling embryos hardly show any sites of DNA-damage. Embryos were imaged using identical camera and illumination settings. The scale bar represents 200 μm. **d** Quantification of the phenotypic fraction of embryos at 2dpf that show a high amount (high) vs. a low amount (low) of sites of DNA damage. The phenotypic fraction is consistent with the expected fraction of 25% as expected from a heterozygous incross of heterozygous *dtymk* fish. The genotype of the low vs. high phenotypic fraction was verified by Sanger sequencing. The number of embryos included in the analysis is *n* = 13 for embryos with high amounts of staining (*dtymk* mutants) and *n* = 38 for sibling embryos showing low to no γH2AX-staining. Two biological replicates produced similar results

As the biosynthesis of one of the nucleotides for DNA synthesis and repair is impaired, one could expect nucleotide pool imbalance in mutant fibroblasts and zebrafish. One of the most common consequences of nucleotide imbalance is the incorporation of ribonucleotides into genomic DNA [36]. Ribonucleotides differ from deoxyribonucleotides by the presence of a single reactive hydroxyl group at the 2’ position of the ribose sugar, making RNA more susceptible to spontaneous hydrolysis [20]. The presence of ribonucleotides in genomic DNA is not favorable as they can impair DNA replication and render DNA more sensitive to strand breakage, leading to genome instability. We evaluated the presence of ribonucleotides in the genomic DNA by alkaline hydrolysis and subsequent alkaline gel electrophoresis (Fig. 5a, b). These experiments show that the genomic DNA isolated from *dtymk –/–* embryos migrates at a lower position and is more fragmented, indicated by a broader smear, in an alkaline agarose gel (Fig. 5a). This indicates a marked increased sensitivity of the genomic DNA of *dtymk –/–* embryos to alkaline hydrolysis reflective of elevated incorporation of ribonucleotides. When compared to the DNA from cells from *Rnaseh2 –/–* mice, a mutant strain known to incorporate high amounts of ribonucleotides in the genome [6, 28], the genomic DNA of *dtymk –/–* zebrafish shows a similar degree of fragility resulting in a broad smear of lower molecular weight and lower intensity (Supplementary Fig. 3c, online resource).

Virtually all DNA repair pathways involve some degree of repair-associated DNA synthesis. The effects of *DTYMK* deficiency on the process of DNA damage signaling was evaluated in vivo. Embryos were UV-irradiated at 24hpf. DNA breakage was visualized by γH2AX staining. The embryos were allowed to recover for 24 h and the number of γH2AX-positive cells, visible as small black dots throughout the embryo, in *dtymk* mutant fish was evaluated (Fig. 5c, d). From a batch of embryos from a heterozygous incross, two distinct populations can be discriminated: one group with high amounts of strongly stained γH2AX cells and another group with no to very low levels of γH2AX-positive cells. The fraction of embryos from a *dtymk* heterozygous incross with high amounts of γH2AX-positive cells was about 25%. Genotyping confirmed that these were the *dtymk* homozygous mutant fraction. This analysis shows elevated levels of γH2AX persisting in the *dtymk* mutant embryos 24 h post UV-irradiation compared to the wild-type (sib) counterparts. This abnormally elevated DNA damage response signaling is consistent with elevated levels of unrepaired DNA breaks in the *dtymk* mutants under these conditions (Fig. 5c, d).

**Fig. 6:**
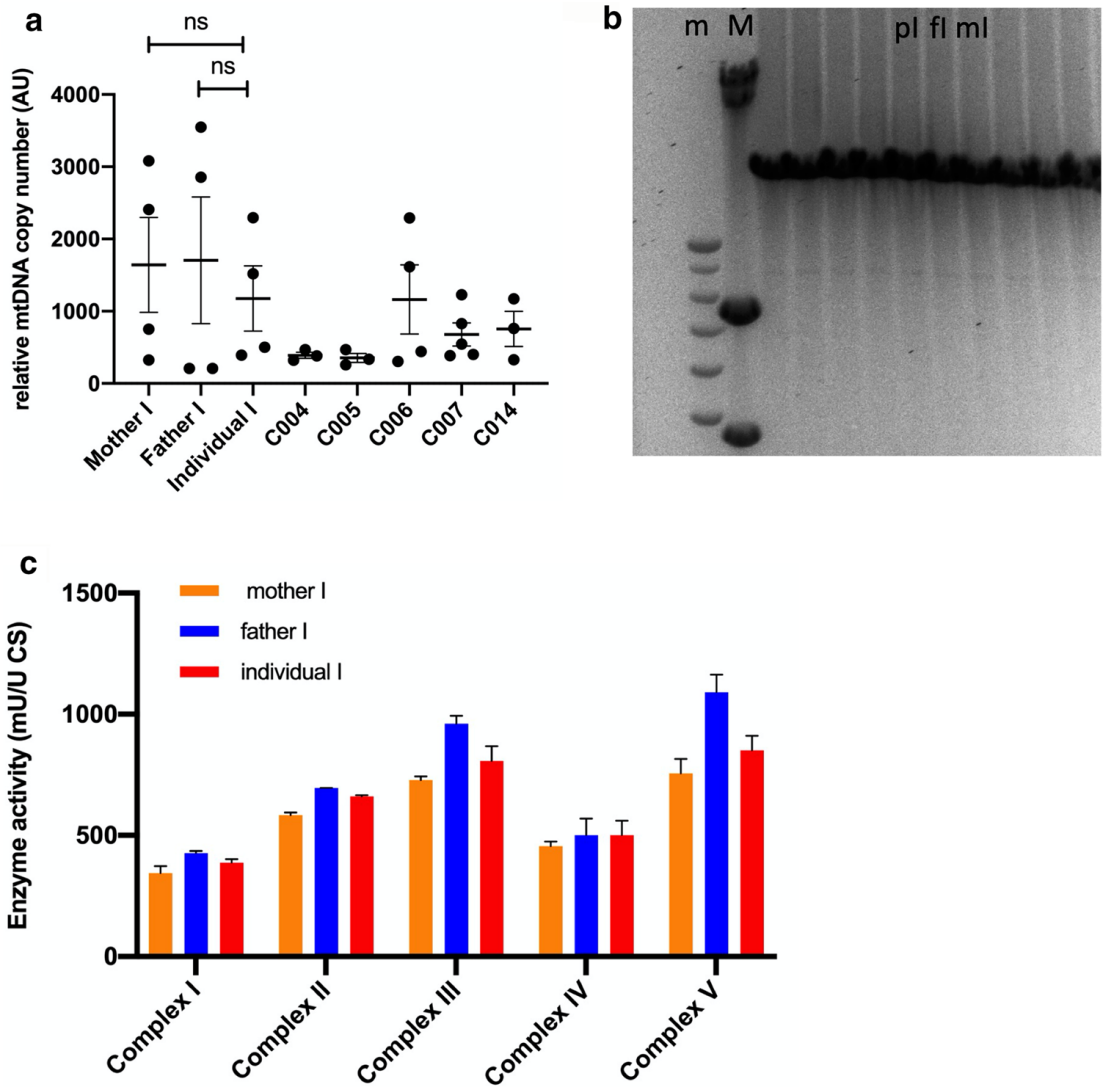
Mitochondrial DNA copy number, integrity and complex activity. **a** Relative mtDNA copy number in fibroblasts of individual I, the parents of individual I and 5 healthy controls. mtDNA copy number was assessed by comparing expression levels of a mitochondrial DNA fragment (*D-loop*) versus a nuclear encoded gene (*B2M*). Relative mtDNA copy number was calculated by calculating Δ*CT* (= *CT* nuclear gene, *B2M*–CT mitochondrial gene, *D-loop*) and converting ΔCT to relative expression levels (relative expression = 2^∆*CT*^) [31]. Results are pooled values from 4 experiments and plotted as average ± SEM. **b** MtDNA integrity by long-range PCR. Lane 1 and 2 contain DNA ladders (m); 1 kb Plus ladder and (M); HindIII-digested λDNA fragments ladder, respectively. Lanes 3–6 contain anonymized diagnostic samples, lane 7 contains the sample of individual I, lane 8 the father of individual I and lane 9 the mother of individual I. Lane 10–12 also contain anonymized diagnostic samples. This procedure is a diagnostic test, performed at Clinical Genetics at Maastricht UMC + . No large mtDNA deletions could be observed. **c** Mitochondrial respiratory complex activity. Enzymatic activity of mitochondrial respiratory complexes I–V were measured using a spectrophotometer in lysates of cultured fibroblasts [33]. Relative values are calculated using the value of Citrate Synthase activity as the reference value. Bars are averages of 2 measurements. This assay is a diagnostic assay, performed at the Translational Metabolic Laboratory of Radboud UMC

In a recent study, *DTYMK* was suggested to cause mitochondrial DNA (mtDNA) depletion [29]. Therefore, we studied the mitochondrial genome and function in cultured fibroblasts of individual I (Fig. 6) and the parents of individual I. For additional reference, we included 5 fibroblast samples of healthy controls. The mtDNA copy number in fibroblasts of individual I was found to be lower than that of the parents but this was not statistically different. Moreover, mtDNA copy number in fibroblasts of the parents of individual I is higher than in control fibroblasts (Fig. 6a) and the mtDNA copy number of individual I is in the same range as the control cells.

Deficiencies in the replication machinery of the mtDNA can also result in deletions in the mtDNA. Therefore, we studied the integrity of the mitochondrial genome by means of long-range PCR, amplifying the complete mitochondrial genome (Fig. 6b). The mitochondrial genome amplified from individual I and its parents migrate at the expected length of 16,5 kilobases and show no indications of mtDNA deletions. We conclude that the mtDNA copy number and integrity are not compromised by *DTYMK* deficiency. Finally, mitochondrial function was assessed. We evaluated enzyme activity of respiratory complexes I–V using a spectrophotometric assay as used in mitochondrial disease diagnostics (Fig. 6c). individual I shows no significant alterations in the enzyme activities of complexes I–V, indicating that mitochondrial respiratory function in individual I is comparable to that of the parents. In conclusion, we find no significant indications of mitochondrial dysfunction in fibroblasts of individual I.

## Discussion

We report loss-of-function of *DTYMK* as the genetic cause of a novel, severe neurodegenerative disease in two unrelated individuals with a dramatic neurodevelopmental decline. Brain pathology confirmed massive neuronal dropout in virtually the entire brain, sparing the brain stem. This striking neurodegenerative phenotype is also observed in *dtymk* mutant zebrafish which exhibit microcephaly, brain edema and neuronal apoptosis (Fig. 3). These results, as well as the cerebral atrophy that only sets in postnatally and the presence of apoptotic cells in the brain of individual I (Fig. 2j) implicate neuronal apoptosis as one of the hallmarks of this disease.

This report is the second study describing *DTYMK* variants in the context of human disease. Lam et al*.* recently reported two brothers with microcephaly, hypotonia and severe intellectual disability showing compound heterozygous variants in *DTYMK* [17]. They predicted mitochondrial depletion in one of the affected individuals, but functional data to support a mitochondrial defect was not provided [17]. In the current study, we present *DTYMK* variants in two unrelated families and provide biochemical data from fibroblasts of affected individuals and an additional animal model. Furthermore, we demonstrate important aspects of the pathogenic mechanism. Although the clinical phenotype of our individuals highly resembles that described previously, we could not document mtDNA depletion. (Fig. 6a). We acknowledge that measurement of mtDNA copy number in fibroblasts (or peripheral blood) is not representative for the copy number in affected tissues, as compensatory mechanisms can mask mtDNA depletion [9, 30]. For diagnostic purposes, mtDNA copy number can only be reliably assessed in a muscle biopsy of affected individuals. Unfortunately, we do not have access to muscle tissue of the described individuals. Still, additional functional studies failed to demonstrate mitochondrial dysfunction. Mitochondrial parameters, mtDNA integrity and respiratory chain activity, were all within the normal range in individual I. Also, all biochemical indicators for mitochondrial dysfunction from our individuals that were measured in serum and urine were normal: lactate and alanine levels were normal and no generalized hyper aminoaciduria was observed.

Several other disease-causing genetic defects in human dTTP metabolism are known. Thymidine phosphorylase (TP) deficiency causes MNGIE, Mitochondrial Neuro Gastro-Intestinal Encephalomyopathy [24]. TP catalyses the cleavage of thymidine into ribose-1-phosphate and thymine (Fig. 1c). When accumulation of dTTP occurs, dNTP pool imbalance is promoted, leading to mtDNA depletion [12]. Inactivating variants in *RRM2B*, encoding part of Ribonucleotide Reductase (RR) causes a severe mtDNA depletion syndrome that is fatal in the neonatal period [4]. RR catalyses the reduction of ribonucleoside diphosphates to deoxyribonucleoside diphosphates in the de novo biosynthetic pathways of all four canonical dNTP. Another human neurodegenerative disease, linked to dTTP metabolism and caused by pathogenic variants in *CAD* (Fig. 1a) is developmental and epileptic encephalopathy 50 (DEE50) [15, 22]. This is an autosomal recessive, progressive neurodegenerative neurometabolic disorder characterized by delayed psychomotor development, early onset seizures, severe developmental regression, and normocytic anemia. Onset is within the first months or years of life [15, 22]. Lastly, DTYMK deficiency resembles Aicardi–Goutières syndrome (AGS), both at the clinical as at the pathophysiological level. AGS is characterized by progressive microcephaly, psychomotor retardation and often death in early childhood [8]. White matter destruction and brain atrophy are shared phenotypic features of AGS and DTYMK deficiency. Clinical differences are that in AGS, intracranial calcification and systemic features (thrombocytopenia, hepatosplenomegaly and cerebrospinal fluid lymphocytosis) are present [2]. Importantly, at the mechanistic level, AGS and DTYMK deficiency are similar as both are characterized by elevated ribonucleotide incorporation in the genome. In AGS, this is explained by pathogenic variants in the genes encoding ribonuclease H2 subunits [8]. Ribonuclease H2 is the key enzyme, involved in ribonucleotide excision repair (RER). Impairment of this pathway results in the increased genomic incorporation of ribonucleotides [8]. Similarly, in DTYMK deficiency, ribonucleotide incorporation is explained by the lack of sufficient postnatal availability of one of the building blocks of DNA, due to a complete block of the biosynthetic pathway of dTTP.

A notable difference between the human phenotype and the *dtymk*-deficient zebrafish model is the degree in which eye development is affected. In both individuals, some degree of visual impairment was noted as the individuals showed no visual tracking of objects and were thought only to be able to distinguish light and dark. However, the eyes were morphologically normal in both individuals. In the zebrafish model, there is a severe eye phenotype consisting of smaller, deformed eyes lacking multiple cellular layers (Supplementary Fig. 2d, online resource). Similar eye phenotypes have been reported in knockout models of other enzymes of nucleotide metabolism [38]. In *perplexed* mutants, which have a pathogenic variant in *cad* (carbamoyl-phosphate synthase 2, aspartate transcarbamylase and dihydroorotase), the enzyme active in the initial step of de novo dTTP synthesis (Fig. 1a), eye defects are similar to *dtymk*-deficient embryos: eyes are small and lack retinal lamination (Supplementary Fig. 2d, online resource) [38]. Other phenotypic features of *perplexed* mutants include absence of the lower jaw cartilage. This cartilage defect is also observed in *dtymk* mutants (Fig. 3c). A second mutant affecting de novo synthesis is hi688, which is defective in ribonucleotide reductase R2 (*rrm2*), is early lethal and leads to a general degeneration phenotype [11, 38]. This phenotype is also reminiscent of the *dtymk* phenotype in which early lethality and general degeneration is observed.

We provide additional insight into the phenotypic presentation of severely compromised dTMPK activity. We show a marked impairment in DNA replication in fibroblasts of an affected individual (Fig. 4c). In addition, we demonstrated significantly reduced cellular proliferation (Fig. 4d) and apoptosis (Fig. 4e, f) in the brain of *dtymk* mutant zebrafish and elevated levels of UV-induced γH2AX suggestive of elevated genomic instability in zebrafish embryos (Fig. 5c, d). Genome instability is most probably caused by ribonucleotide incorporation in the genome (Fig. 5a, b). Ribonucleotide incorporation is one of the most abundant forms of DNA damage in eukaryotic cells, making the DNA more fragile [21, 23, 36]. Therefore, a repair mechanism: RER, is in place to remove ribonucleotides from genomic DNA. A critical component of RER is *RNaseH2* [19]. Mouse mutants for *RNaseH2* accumulate large numbers of ribonucleotides in their genomic DNA resulting in genome instability, arrest in cellular proliferation and a p53-dependent DNA-damage response [6, 28]. We show that *dtymk* mutant zebrafish exhibit elevated incorporation of ribonucleotides in the genomic DNA (Fig. 5a, b). When compared to genomic DNA of *Rnaseh2 –/–* mice, the *dtymk –/–* genomic DNA shows as similar behavior on alkaline gels: a broad smear of lower molecular weight and reduced intensity due to increased degradation (Supplementary Fig. 3c, online resource). The elevated accumulation of ribonucleotides in genomic DNA associated with impaired DNA replication, reduced cellular proliferation and elevated DNA damage response signaling represents a plausible explanation for the severe phenotypic presentation and the early embryonic lethality (40% death at 5dpf) observed in *dtymk* mutant zebrafish. This scenario may contribute to the progressive loss of cerebral cortex tissue in the individuals carrying *DTYMK* variants [1, 34].

It is remarkable that an apparently complete *DTYMK* deficiency is compatible with life. Due to a complete block in the metabolic pathway for dTTP synthesis, an essential building block of DNA cannot be generated. There is no known alternative biochemical pathway that could provide an adequate supply of dTTP to sustain DNA replication and repair as both known pathways (de novo and salvage pathway) converge before the defect in *DTYMK* (Fig. 1a). This raises two outstanding questions: First, what is the compensation mechanism allowing fetal brain development and survival until birth without the ability to generate dTTP? Second, why is the brain most affected? One possibility is that the missense alleles described in the two families in this study still show minimal residual activity, sufficient for very limited DNA synthesis and repair. On the other hand, the zebrafish model is a frameshift allele giving rise to a grossly truncated protein with almost undetectable enzyme activity. The phenotypic similarity of this loss-of-function allele in the zebrafish to that of the affected individuals coupled to the fact that both models show almost undetectable dTMPK enzyme activity strongly suggest that the human variants are loss-of-function variants.

The fact that both in fibroblast as in zebrafish models, dTTP nucleotide levels resemble those of normal controls (Supplementary Fig. 3a, b, online resource) together with the fact that both affected individuals and the homozygous zebrafish survive beyond birth could suggest the intriguing possibility of a compensatory pathway for dTTP generation. A prima facie candidate enzyme would be *TMPK2 (or UMP-CMP kinase 2, CMPK2*) [7, 39]. However, as this enzyme has no apparent capability to use dTMP as a substrate [5, 7, 39] it would appear unlikely that CMPK2 can fulfill this function. Thus, a compensatory pathway for dTTP generation remains to be proven.

It is known that imbalanced dNTP pools cause replication stress that can in turn trigger neuronal apoptosis [25]. Widespread apoptosis is what was observed in the brains of our affected individuals, and in the zebrafish. Why neurons are particularly vulnerable to this imbalance is not clear at this time. A restricted expression pattern of *DTYMK* is unlikely to be the answer as *DTYMK* is expressed in all human tissues. Cerebral cortex, hippocampus and caudate nucleus show medium expression, while cerebellum has high protein expression (https://www.proteinatlas.org/ENSG00000168393-DTYMK/tissue). Interestingly, expression in brain mouse embryo (E15) (http://www.informatics.jax.org/marker/MGI:108396) shows highest *Dtymk* expression levels in the forebrain, moderate levels in the midbrain (tectum) and low expression in the hindbrain. This might provide a clue to why the forebrain-derived structures (cerebral cortex and basal ganglia) are most affected in the described individuals. Alternatively, a restricted expression pattern of a putative compensatory enzyme could explain the vulnerability of the brain in this disease.

In conclusion, we show that novel germline variants in *DTYMK* cause profound postnatal neurodegeneration and marked developmental arrest. Our studies directly link thymidine biosynthesis to genome integrity and postnatal neuronal viability.

## Supplementary Information

Below is the link to the electronic supplementary material.

Supplementary file1 Supplementary Figure 1 (a) Schematic representation of PCR strategy showing efficacy of dtymk morpholinos at 5dpf. A fragment containing exons 1-4 (green arrows) was amplified from cDNA of dtymk-morpholino-injected or control-morpholino-injected embryos and analyzed upon electrophoresis. The binding sites of the morpholinos targeting exon 2 (2i2) and exon 3 (3i3) are indicated in red. NIC: non-injected control, Mis= mismatch (control) morpholino, loading = loading control (galt). Primer sequences are given in Supplementary table 1, online resource. (b) Phenotype at 5dpf of embryos injected with mismatch control (MisMO) morpholino or dtymk-targeting morpholinos (MO2i2 and MO3i3). Panels show representative embryos, photographed in side view (left panels) or dorsal view (right panels). Morpholino sequences are provided in Supplementary table 1, online resource. (c) Quantification of head size in morpholino-injected embryos at 5dpf. Average head size of 17-26 embryos at 5dpf. Head size was estimated by measuring the size of the eye in pixels as in Figure 3d. Low (1ng/ embryo) and high doses (6ng/embryo) of the MOs were injected. MisMO low vs MO2i2 low p=0,0002 (unequal variance); MisMO high vs. 2i2MO high p<0,0001.(d) dTMPK activity of morpolino-injected embryos at 5dpf. Pools of 20-50 embryos, injected with 1ng (low) or 6ng (high) dose of mismatch control (MisMO) or dtymk morpholino (MO2i2) were analyzed for dTMPK activity, as in Figure 4. The potential effect of injection was studied by analysis of non-injected control (NIC) embryos. (TIFF 28567 KB)

Supplementary file2 Supplementary Figure 2 (a) Comparison of the outcomes of the most important variant interpretation tool predictions for both DTYMK variants, p.(Pro81Leu) and p.(Asp128Asn) and their predicted location in the 3D protein structure as modelled using HOPE [32, 35]. (b) Quantification of the dtymk -/- phenotype at 5dpf. n indicates the number of embryos with the depicted phenotype from mass matings of heterozygous mutant dtymk fish. A fraction of 25% is expected to be homozygous mutants. The fraction carrying the normal phenotype is indicated by the blue bar whereas the mutant phenotype is marked by an orange or red bar, depending on the absence or presence of brain edema, respectively. Phenotypes are illustrated by representative images of the head of genotyped embryos at 5dpf. (c) Length of dtymk mutant and sibling embryos at 2dpf. Length is not significantly different between both groups (p=0.44). 19-24 embryos/group were measured. Two biological replicates.(d) Detail of histology of the eye of sibling (sib) and homozygous dtymk mutant (dtymk -/-) embryos at 3dpf. Note the absence of all retinal cell layers, except for the RPE and PRL in the dtymk -/- embryos. RPE: retinal pigmented epithelium; PRL: Photoreceptor layer; OPL: outer plexiform layer; INL: inner nuclear layer; IPL: inner plexiform layer; GCL: ganglion cell layer. The scale bar represents 100 m.(e) Representative images of dorsal view images of embryos at 2dpf showing sites of DNA-damage repair, visualized by H2AX-staining (black dots). The mutant embryos clearly show many sites of unrepaired DNA-damage whereas the sibling embryos hardly show any sites of DNA-damage. Embryos were imaged using identical camera and illumination settings. The scale bar represents 200 m. (f) Upper left panel: Human TMPK structure was extracted from the Protein Data Bank (pdb code: 1e2f), and is shown with bound ligands (dTMP and ATP analog shown as spheres). Residue P81 was labeled in purple and D128 in red. The N- and C-terminus were labeled in slate. Residues P81 (purple) and D128 (red) are located far away from the ligand binding site. Upper right panel: The structure was turned 180°. Both residues (P81 and D128) are shown. Lower left panel: Detailed view of residue P81 and its interactions. Lower right panel: Detailed view of residue D128, which is located at the bottom of a β sheet, which forms the active site, close to the N-terminus. All the interacting residues are labeled in pink and are important for stability of the β sheet. (TIFF 28567 KB)

Supplementary file3 Supplementary Figure 3(a) Quantification of dNTP pools in fibroblasts. The quantity of canonical nucleotides (dATP, dGTP, dTTP, dCTP) was measured in fibroblasts of individual I and both parents. Values are averages of 6 measurements.(b) Quantification of dNTP pools in sibling and dtymk mutant larvae at 5dpf. Values are averages of 3 measurements.(c) Representative image of alkaline gel electrophoresis of dtymk -/- zebrafish and Rnaseh2 -/- mouse embryonic fibroblasts (MEF). Genomic DNA (850ng) of dtymk mutant embryos (dtymk -/-) and sibling (sibs) embryos at 5dpf was subjected to alkaline gel electrophoresis and compared to 500ng of genomic DNA from Rnaseh2 -/- MEF and Rnaseh2 +/+ MEF on the same gel. The first and last lane contain the marker Generuler 1kb Plus DNA ladder (Thermo Fisher). Three biological replicates produced highly similar results. (TIFF 28567 KB)

Supplementary file4 Supplementary Figure 4(a) Overview of the pyrimidine biosynthesis pathway (WikiPathways; https://www.wikipathways.org/index.php/Pathway:WP4022#nogo2) with focus on dCTP, dUTP and dTTP. Genes for which expression analysis was performed are indicated by red circles. (b) Relative expression levels of CMPK1, CMPK2, DTYMK and TK1 in fibroblasts of members of family I, compared to healthy controls using RT-QPCR. Relative expression is calculated using the 2−ΔCT method using B2M as a reference (housekeeping) gene. For normalization, average expression levels in 3 different control fibroblast lines were used as reference values. Results are plotted as fold change from three biological replicates, measured in 2-3 fold. The dotted line represents the average expression level of the studied genes in control fibroblasts. Expression of an additional housekeeping gene, GAPDH, was added to illustrate a low degree of variation in genes unrelated to nucleotide metabolism. (TIFF 28567 KB)

Supplementary file5 (DOCX 27 KB)

Supplementary file6 (DOCX 13 KB)
