# Supplementary figures and images for: DTYMK is essential for genome integrity and neuronal survival
Source: Acta Neuropathol. 2021 Dec 17;143(2):245–62. doi: 10.1007/s00401-021-02394-0 (PMC8742820; doi:10.1007/s00401-021-02394-0)

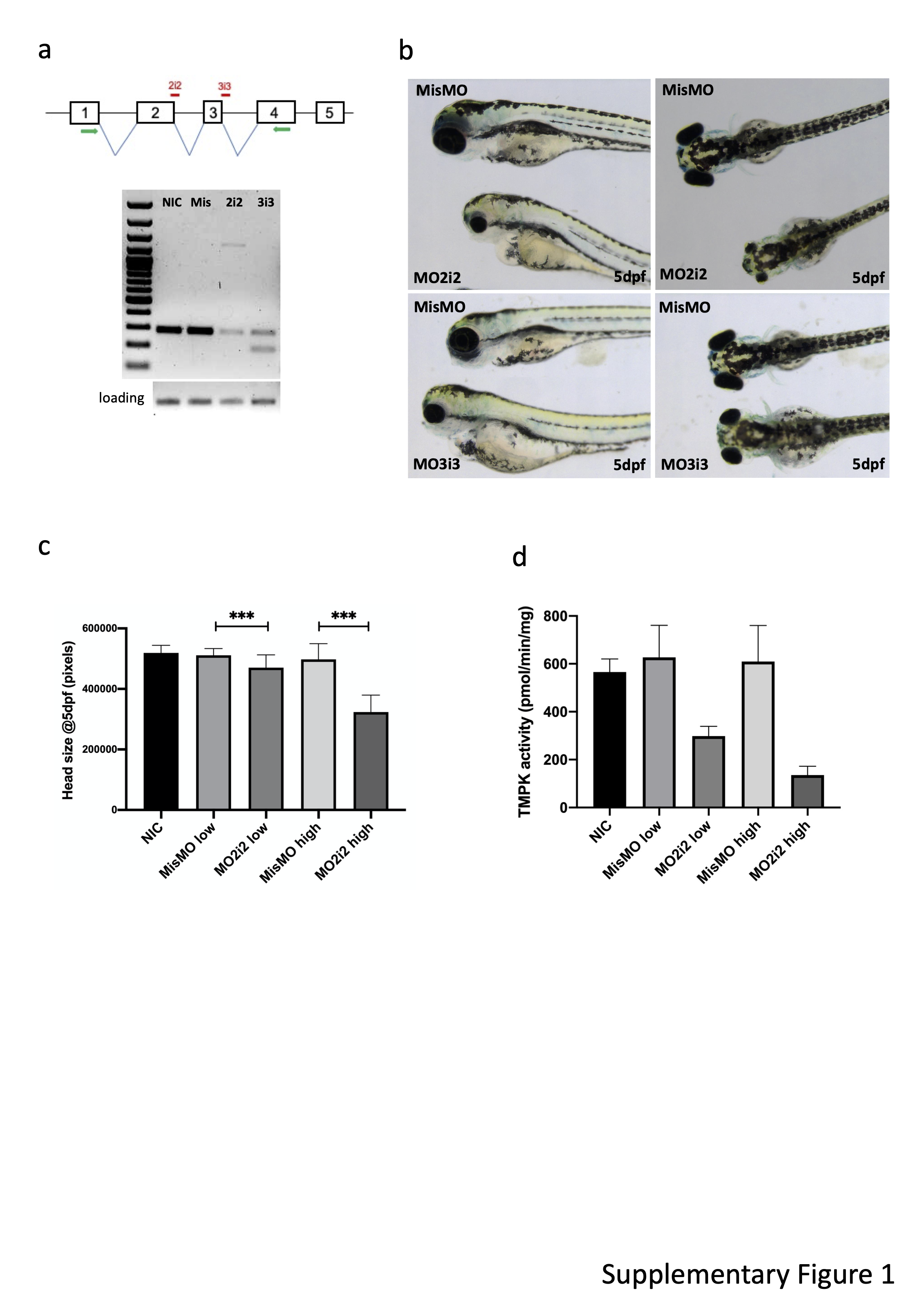

Supplement: Supplementary file 1 — Supplementary file1 Supplementary Figure 1 (a) Schematic representation of PCR strategy showing efficacy of dtymk morpholinos at 5dpf. A fragment containing exons 1-4 (green arrows) was amplified from cDNA of dtymk-morpholino-injected or control-morpholino-injected embryos and analyzed upon electrophoresis. The binding sites of the morpholinos targeting exon 2 (2i2) and exon 3 (3i3) are indicated in red. NIC: non-injected control, Mis= mismatch (control) morpholino, loading = loading control (galt). Primer sequences are given in Supplementary table 1, online resource. (b) Phenotype at 5dpf of embryos injected with mismatch control (MisMO) morpholino or dtymk-targeting morpholinos (MO2i2 and MO3i3). Panels show representative embryos, photographed in side view (left panels) or dorsal view (right panels). Morpholino sequences are provided in Supplementary table 1, online resource. (c) Quantification of head size in morpholino-injected embryos at 5dpf. Average head size of 17-26 embryos at 5dpf. Head size was estimated by measuring the size of the eye in pixels as in Figure 3d. Low (1ng/ embryo) and high doses (6ng/embryo) of the MOs were injected. MisMO low vs MO2i2 low p=0,0002 (unequal variance); MisMO high vs. 2i2MO high p<0,0001.(d) dTMPK activity of morpolino-injected embryos at 5dpf. Pools of 20-50 embryos, injected with 1ng (low) or 6ng (high) dose of mismatch control (MisMO) or dtymk morpholino (MO2i2) were analyzed for dTMPK activity, as in Figure 4. The potential effect of injection was studied by analysis of non-injected control (NIC) embryos. (TIFF 28567 KB) [file 401_2021_2394_MOESM1_ESM.tiff]

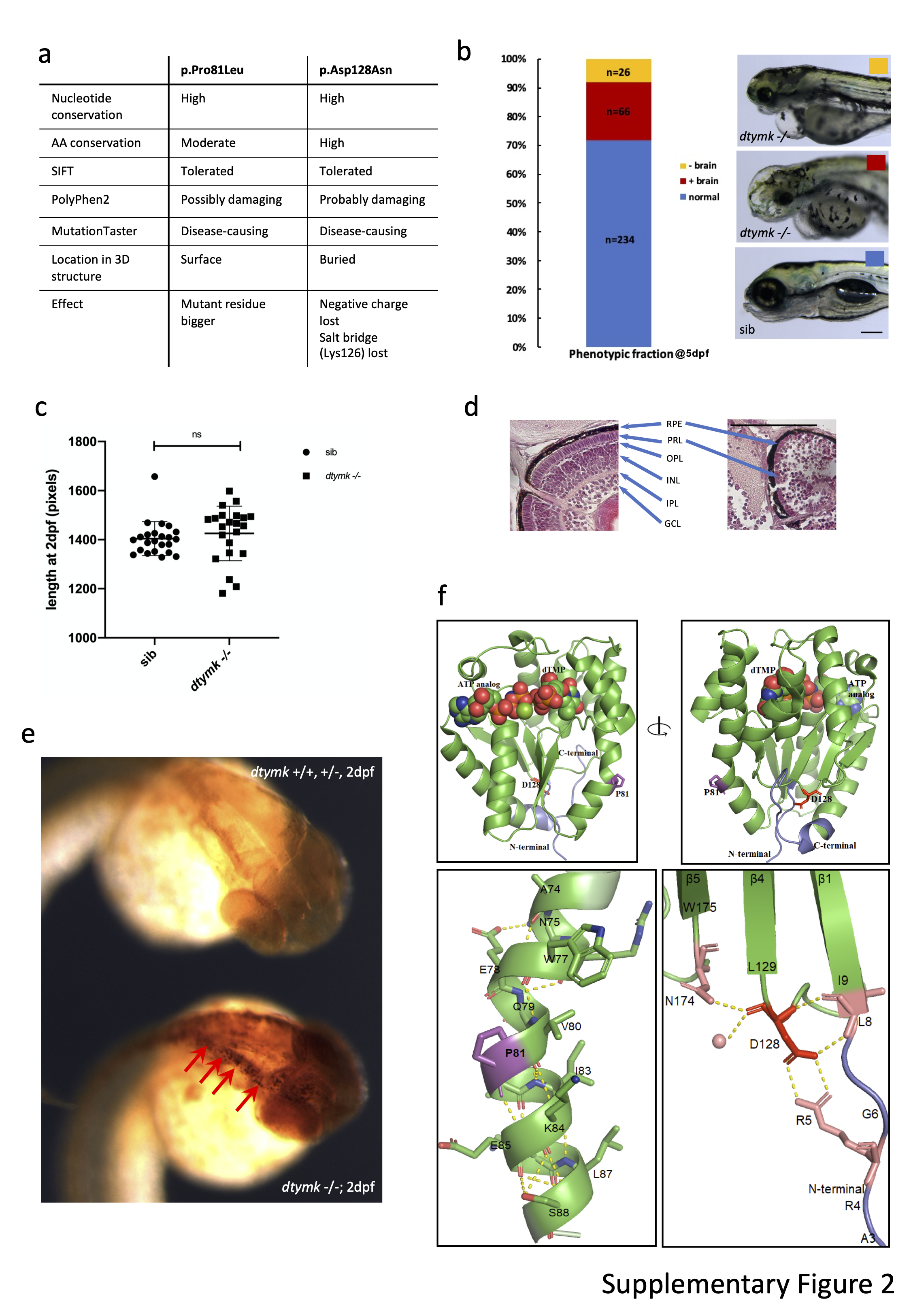

Supplement: Supplementary file 2 — Supplementary file2 Supplementary Figure 2 (a) Comparison of the outcomes of the most important variant interpretation tool predictions for both DTYMK variants, p.(Pro81Leu) and p.(Asp128Asn) and their predicted location in the 3D protein structure as modelled using HOPE [32, 35]. (b) Quantification of the dtymk -/- phenotype at 5dpf. n indicates the number of embryos with the depicted phenotype from mass matings of heterozygous mutant dtymk fish. A fraction of 25% is expected to be homozygous mutants. The fraction carrying the normal phenotype is indicated by the blue bar whereas the mutant phenotype is marked by an orange or red bar, depending on the absence or presence of brain edema, respectively. Phenotypes are illustrated by representative images of the head of genotyped embryos at 5dpf. (c) Length of dtymk mutant and sibling embryos at 2dpf. Length is not significantly different between both groups (p=0.44). 19-24 embryos/group were measured. Two biological replicates.(d) Detail of histology of the eye of sibling (sib) and homozygous dtymk mutant (dtymk -/-) embryos at 3dpf. Note the absence of all retinal cell layers, except for the RPE and PRL in the dtymk -/- embryos. RPE: retinal pigmented epithelium; PRL: Photoreceptor layer; OPL: outer plexiform layer; INL: inner nuclear layer; IPL: inner plexiform layer; GCL: ganglion cell layer. The scale bar represents 100 m.(e) Representative images of dorsal view images of embryos at 2dpf showing sites of DNA-damage repair, visualized by H2AX-staining (black dots). The mutant embryos clearly show many sites of unrepaired DNA-damage whereas the sibling embryos hardly show any sites of DNA-damage. Embryos were imaged using identical camera and illumination settings. The scale bar represents 200 m. (f) Upper left panel: Human TMPK structure was extracted from the Protein Data Bank (pdb code: 1e2f), and is shown with bound ligands (dTMP and ATP analog shown as spheres). Residue P81 was labeled in purple and D128 in red [file 401_2021_2394_MOESM2_ESM.tiff]

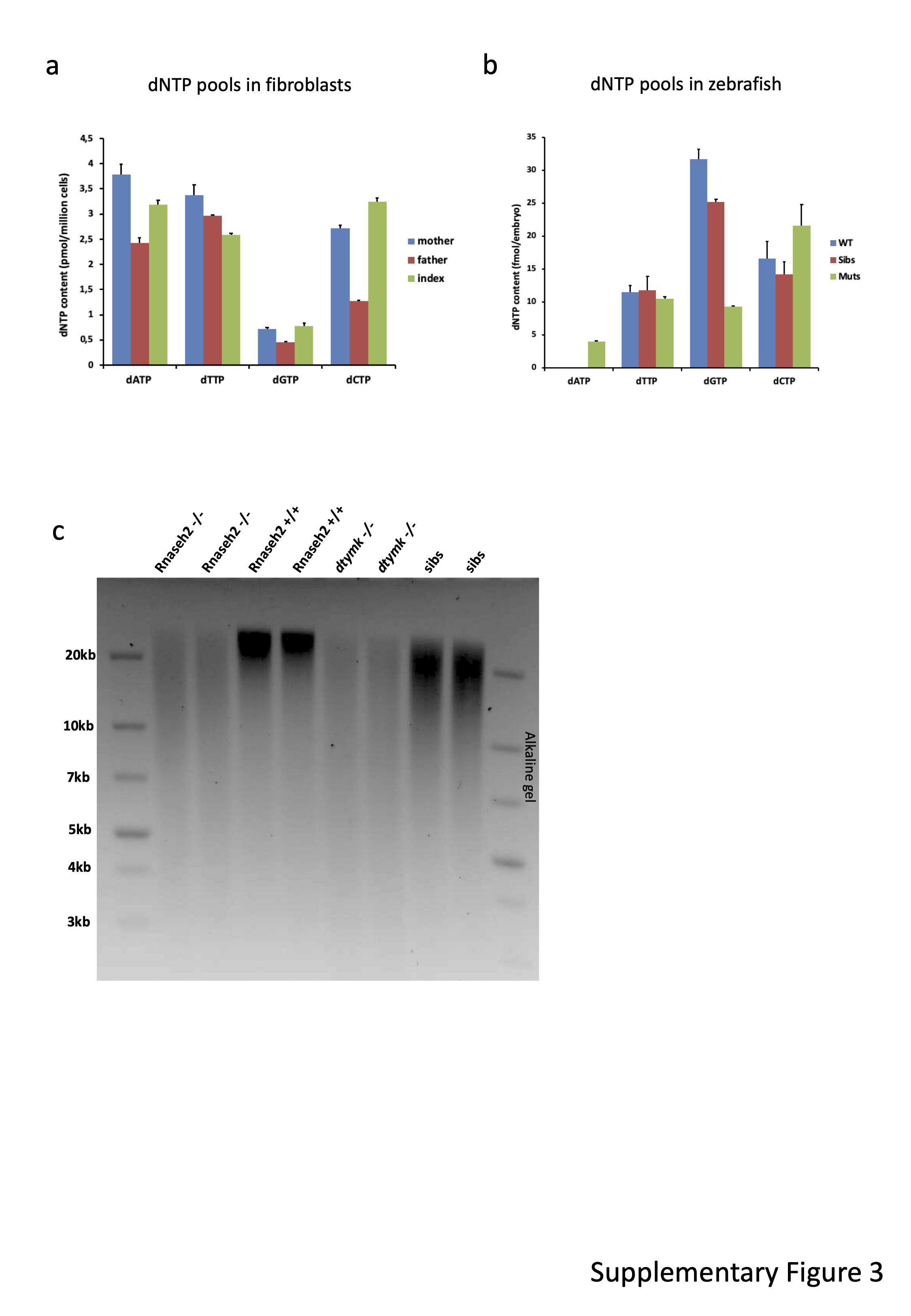

Supplement: Supplementary file 3 — Supplementary file3 Supplementary Figure 3(a) Quantification of dNTP pools in fibroblasts. The quantity of canonical nucleotides (dATP, dGTP, dTTP, dCTP) was measured in fibroblasts of individual I and both parents. Values are averages of 6 measurements.(b) Quantification of dNTP pools in sibling and dtymk mutant larvae at 5dpf. Values are averages of 3 measurements.(c) Representative image of alkaline gel electrophoresis of dtymk -/- zebrafish and Rnaseh2 -/- mouse embryonic fibroblasts (MEF). Genomic DNA (850ng) of dtymk mutant embryos (dtymk -/-) and sibling (sibs) embryos at 5dpf was subjected to alkaline gel electrophoresis and compared to 500ng of genomic DNA from Rnaseh2 -/- MEF and Rnaseh2 +/+ MEF on the same gel. The first and last lane contain the marker Generuler 1kb Plus DNA ladder (Thermo Fisher). Three biological replicates produced highly similar results. (TIFF 28567 KB) [file 401_2021_2394_MOESM3_ESM.tiff]

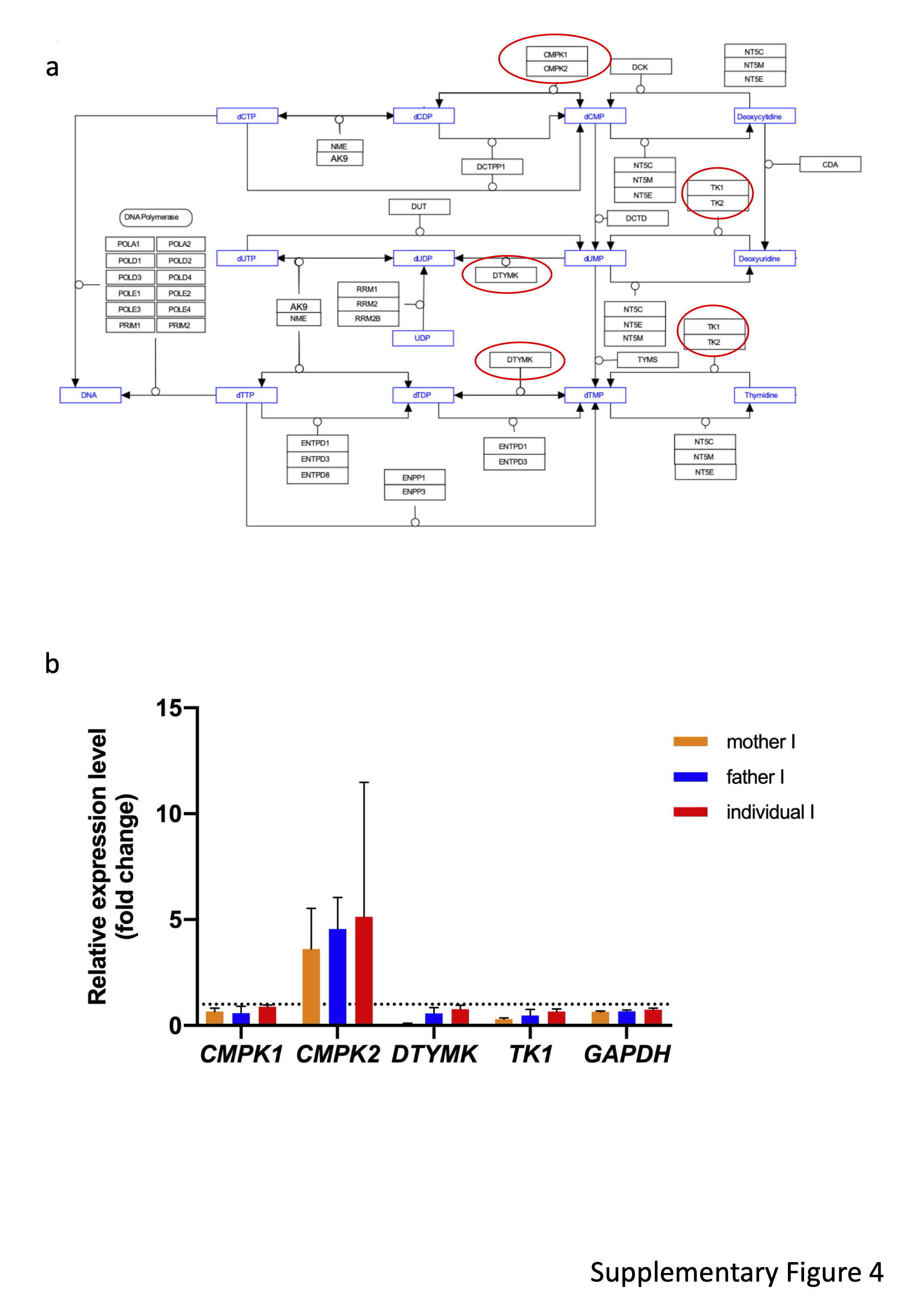

Supplement: Supplementary file 4 — Supplementary file4 Supplementary Figure 4(a) Overview of the pyrimidine biosynthesis pathway (WikiPathways; https://www.wikipathways.org/index.php/Pathway:WP4022#nogo2) with focus on dCTP, dUTP and dTTP. Genes for which expression analysis was performed are indicated by red circles. (b) Relative expression levels of CMPK1, CMPK2, DTYMK and TK1 in fibroblasts of members of family I, compared to healthy controls using RT-QPCR. Relative expression is calculated using the 2−ΔCT method using B2M as a reference (housekeeping) gene. For normalization, average expression levels in 3 different control fibroblast lines were used as reference values. Results are plotted as fold change from three biological replicates, measured in 2-3 fold. The dotted line represents the average expression level of the studied genes in control fibroblasts. Expression of an additional housekeeping gene, GAPDH, was added to illustrate a low degree of variation in genes unrelated to nucleotide metabolism. (TIFF 28567 KB) [file 401_2021_2394_MOESM4_ESM.tiff]
