# Supplementary material for: DTYMK is essential for genome integrity and neuronal survival
Source: Acta Neuropathol. 2021 Dec 17;143(2):245–62. doi: 10.1007/s00401-021-02394-0 (PMC8742820; doi:10.1007/s00401-021-02394-0)
Supplement: Supplementary file 5 — Supplementary file5 (DOCX 27 KB) [file 401_2021_2394_MOESM5_ESM.docx]

**Supplementary Methods**

**Morpholino experiments**

Two splice-blocking Morpholinos were designed and manufactured by GeneTools, targeting exon 2 (2i2) and exon 3 (3i3) of zebrafish *dtymk.* Additionally, mismatch control morpholinos were also designed. Morpholinos were injected in low (1ng/embryo) or high dose (6ng/embryo) in 1-4 cell-stage embryos. Phenotypes were documented and quantified at 5dpf. Splice-blocking action of the morpholinos was confirmed by PCR showing aberrant splicing and reduced expression.

**dNTP pools**

Total cellular nucleotides were extracted with 60% methanol following the protocol published by Martı *et al.* (Marti *et al.*, 2012). Briefly, 2 ml cold methanol (-20°C) was added to each sample, mixed well and then kept at -20°C for 2 hours. The samples were then centrifuged at 16000 x g for 20 min at 4°C. The supernatants were transferred to new tubes and heat-treated (100°C) for 3 min and centrifuged for 20 min at 4°C. The supernatants were transferred to new tubes and dried in a speed Vac. The nucleotides were dissolved in 100 µl H2O and used directly for dNTP measurement using the DNA polymerase assay method essentially as described [1]. The results are given as pmol/million cells of pmol/embryo and presented as mean ± SD of three measurements.

Lysates were prepared from pools of 35-50 larvae of indicated genotypes of cell pellets of cultured fibroblasts.

**Expression analysis**

**﻿**RNA was extracted from fibroblasts using TRIzol (Life Technologies) and converted into cDNA using the qScript cDNA Synthesis Kit (Quantabio). Quantitative real-time PCR (qPCR)-based was performed using SYBR® green (Bioline) on a LightCycler ®480 (Roche Applied Science) apparatus. Relative expression values were calculated using the 2^-ΔΔCt method [2] using *B2M* as a reference gene and expression levels of control fibroblasts as reference levels. Primer sequences are available on request. Samples, obtained from 3 independent RNA extractions were measured in 2-3 fold.

Supplemental references

2. Livak KJ, Schmittgen TD (2001) Analysis of relative gene expression data using real-time quantitative PCR and the 2-ΔΔCQ method. Methods. 25:402–8.

1. Martí R, Dorado B, Hirano M (2012) Measurement of mitochondrial dNTP pools. Methods Mol. Biol. *837*, 135-48.
