# Supplementary material for: DTYMK is essential for genome integrity and neuronal survival
Source: Acta Neuropathol. 2021 Dec 17;143(2):245–62. doi: 10.1007/s00401-021-02394-0 (PMC8742820; doi:10.1007/s00401-021-02394-0)
Supplement: Supplementary file 6 — Supplementary file6 (DOCX 13 KB) [file 401_2021_2394_MOESM6_ESM.docx]

**Supplementary Table**

**Primer sequences**

HRMtmpk_exon4For TTTCAGGGCTTTTCTCTGGA

HRMtmpk_exon4Rev ACGTTGGAAAGCACTCGTCT

DTYMK_05_F tgtaaaacgacggccagtTCTCCTGACCTTGTGATCCG

DTYMK_05_R caggaaacagctatgaccGTAYTCGCATTCAAGGGCAG

zfdtymk_ex4For tgtaaaacgacggccagtGTCCACCCATCTGTCAGTCA

zfdtymk_ex4Rev caggaaacagctatgaccTCACCTTCCAGTTGATTGAGGT

MM_16426_NGS_F CCGCACAAGAGTGCTACTCTCCTC

MM_16425_NGS_R GATATTGATTTCACGGAGGATGGTG

MOdtymkEx1-4For CGGCAGAAATTATGCGATTT

MOdtymkEx1-4Rev GACCAGGTCTGGTTTTGGAA

Loading_galt_ex3_F ATCGTTTGAAGCCAAAATCG

Loading_galt_ex3_R TGCGTATTTCTCTGGATTTGC

**Morpholino sequences**

dtymkMismatchControl (MismMO) ACCGTTCAAAAAACACCTAGACCAT

dtymkSBe2i2 (MO2i2) ACCCTTGAATAAAGACCTACACCAT

dtymkSBe3i3 (MO3i3) TGAAATATTGAACCACTTACAGGCT

**Antibodies**

anti-γH2AX (gamma H2A histone family member X) (GeneTex GTX127342)

anti-phospho-Histone H3 (Ser10) (pH3) (Millipore 06-570).
